# In silico identification of effector proteins from generalist herbivore *Spodoptera litura*

**DOI:** 10.1186/s12864-020-07196-4

**Published:** 2020-11-23

**Authors:** Vinod Kumar Prajapati, Mahendra Varma, Jyothilakshmi Vadassery

**Affiliations:** 1 National Institute of Plant Genome Research (NIPGR), Aruna Asaf Ali Marg, New Delhi, 110067 India; 2 Present Address-Population Ecology Group, Institute of Ecology and Evolution, Friedrich Schiller University Jena, Dornburger Straße 159, 07743 Jena, Germany

**Keywords:** *Spodoptera litura*, Effector proteins, de novo transcriptomics, Herbivory, Plant defense

## Abstract

**Background:**

The common cutworm, *Spodoptera litura* Fabricius is a leaf and fruit feeding generalist insect of the order Lepidoptera and a destructive agriculture pest. The broad host range of the herbivore is due to its ability to downregulate plant defense across different plants. The identity of *Spodoptera litura* released effectors that downregulate plant defense are largely unknown. The current study aims to identify genes encoding effector proteins from salivary glands of *S. litura* (Fab.).

**Results:**

Head and salivary glands of *Spodoptera litura* were used for *de-novo* transcriptome analysis and effector prediction. Eight hundred ninety-nine proteins from the head and 330 from salivary gland were identified as secretory proteins. Eight hundred eight proteins from the head and 267 from salivary gland proteins were predicted to be potential effector proteins.

**Conclusions:**

This study is the first report on identification of potential effectors from *Spodoptera litura* salivary glands.

**Supplementary Information:**

The online version contains supplementary material available at 10.1186/s12864-020-07196-4.

## Background

*Spodoptera litura* Fabricius (Order-Lepidoptera) or common cutworm is one of the most destructive insect pest of agricultural crops, with more than 120 host plants globally [1]. They are polyphagous defoliators which have detrimental effect on cotton, flax, groundnut, jute, maize, rice, soybean, tea, tobacco, and vegetables throughout the tropical and temperate Asia [2]. *S. litura* is responsible for 10–30% reduction in the yield of various crops due to vigorous defoliation and fruit feeding [3]. Related species, *Spodoptera frugiperda* (Fall armyworm) is a devastating pest and has caused USD 3–6 billion annual damage to maize and other African food staples [4].

As they feed, caterpillars use oral secretion (OS) to transport the chewed leaf tissues into their mouth. Oral secretions are produced by salivary glands of insects. Oral secretion includes regurgitant, labial and mandibular saliva that provides a milieu of elicitors/ HAMPs (herbivore associated molecular patterns) and the effectors of which some are recognized by the plants and modulates the plant defense [5]. Variety of HAMPs have been identified and analyzed in the oral secretion of chewing insects, including fatty acid-amino acid conjugates (e.g., volicitin), long-chain α,ω-diols (e.g., bruchins), and plant-derived peptides named inceptins [5]. The HAMPs/elicitors activate plant defense via jasmonate signaling [6]. Insects also produce an array of effector proteins, which make them more virulent against plants by suppressing the plant defense mechanism at multiple steps. It is known that *S. littoralis* oral secretion contains unidentified effectors that suppress systemic Ca^2+^ elevation and wound-induced responses, like the expression of ERF transcription factor [7, 8].

In sucking insect clade, many effectors have been identified. Expressed sequence tag (EST) libraries were used to predict candidate effector proteins from pea aphid *Acyrthosiphon pisum* and green peach aphid, *Myzus persicae* [9, 10]*.* Salivary-secretory proteins from *A. pisum,* and whitefly, *B. tabaci* were identified using a dual transcriptome-proteome based approach [9, 11]. *In-silico* effector prediction has been performed on salivary gland transcriptome of various insects such as Potato leaf hopper, *Empoasca fabae* [12], brown plant hopper, *Nilaparvata lugens* [13], Rice green planthopper, *Nephotettix cincticeps* [14] and grain aphid, *Sitobia avenue* [15]. NlSEF1 and endo β 1–4 endoglucanase effector proteins were identified in the oral secretion of brown plant hopper, *N. lugens.* NlSEF1 is a calcium-binding protein that suppresses the plant defense induced cytosolic Ca^2+^ elevation [16]. Endo β 1–4 endoglucanase effector degrades cellulose in the rice cell wall, resulting in decreased cell wall defense [17]. Whitefly, *B. tabaci* Bt56 effector protein affects salicylic acid (SA) elevation [18]. Effector Mp10 from peach aphid, *Myzus persicae* reduces fecundity, whereas effector MpC002 increases fecundity on *N. benthamiana* [10]. Overexpression of salivary proteins Te28, Te84 and Tu28, Tu84 from spider mites *Tetranychus evansi* and *T. urticae,* respectively suppress SA and increase *T.urticae* ovipostion [19].

Glucose oxidase (GOX) was the first effector protein identified from chewing insect *Helicoverpa zea* saliva, a salivary secretion that suppresses nicotine production in *Nicotiana benthamiana* [20]. ATPase from *Helicoverpa zea* is an ATP hydrolyzing enzyme that suppresses defense activated genes in tomato [21]. Recently, HARP1 (*H. armigera* R- like proteins 1) was characterized as an effector protein in cotton bollworm, *Helicoverpa armigera* [22]. HARP1 interacts with JASMONATE-ZIM-domain (JAZ) proteins, the suppressors of jasmonate (JA) pathway, and blocks signal transduction by preventing JAZ degradation [22].

No comprehensive study has been done to identify effector proteins secreted from salivary glands of the pest insect, *S. litura*. We hypothesize that a generalist insect such as *S. litura* can parasitize wide plant hosts by evading the plant immune system and effectors proteins, secreted by the insect into plant cells, are responsible for this ability. In this study, we used *in-silico* effector prediction pipeline to identify the effector protein-encoding genes from *S. litura* salivary glands.

## Results

### Illumina sequencing and de novo transcriptomics assembly

**Fig. 1:**
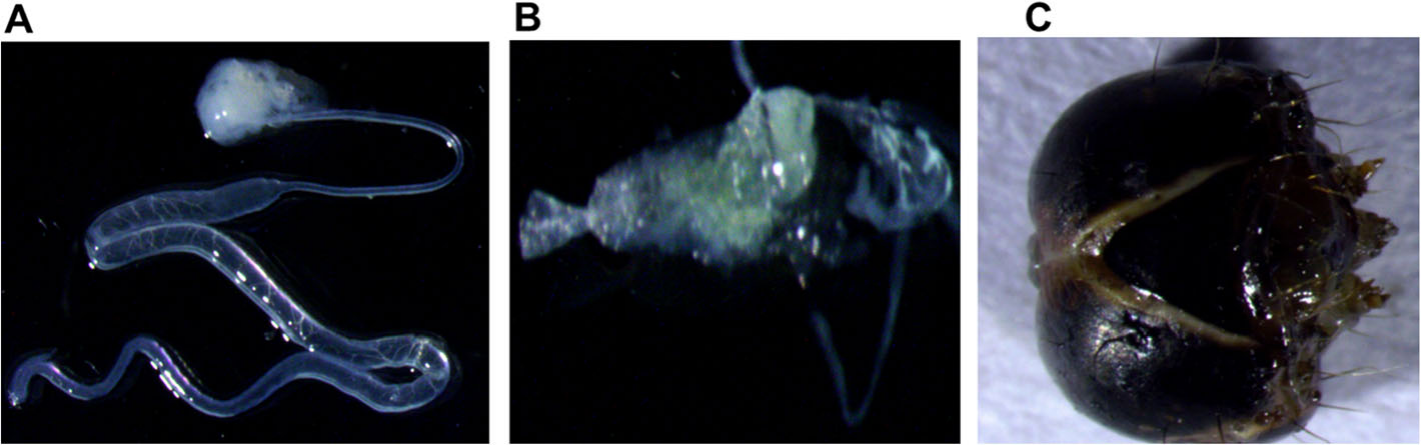
Salivary glands present in *S. litura* after dissection **a**) Labial salivary gland **b**) Ventral eversible gland **c**) Head

**Fig. 2:**
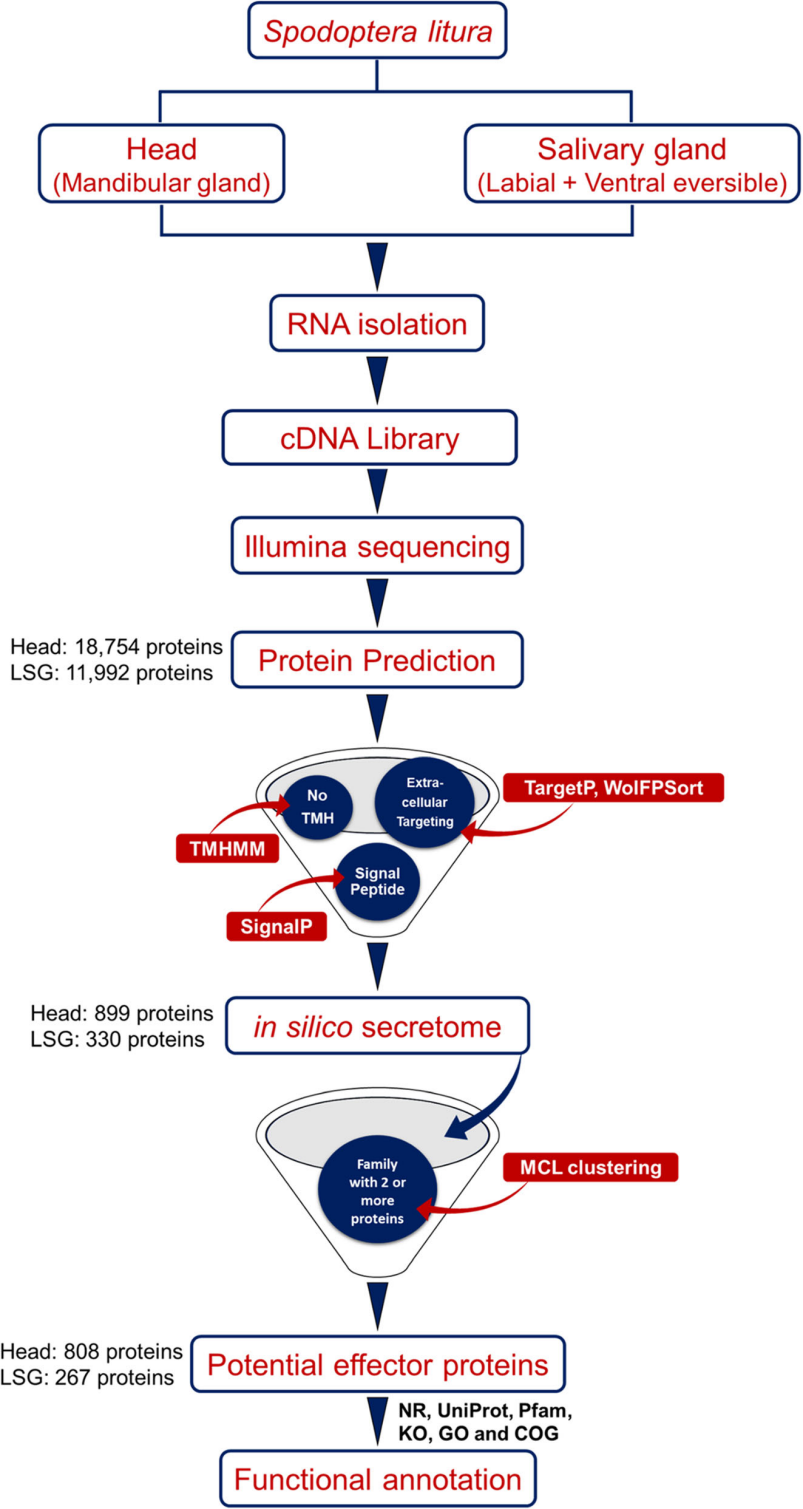
Overview of in silico pipeline to identify potential effector proteins from generalist herbivore, *Spodoptera litura. Spodoptera litura* 4th instar larvae were used for salivary gland isolation. RNA was isolated from two samples; head (mandibular glands) and salivary gland (LSG and VEG) and was sequenced using Illumina platform. Raw reads were processed and later four filtering steps; presence of signal peptide, no transmembrane helices (TMH), extracellular targeting and clustering into families were sequentially applied to obtain a database of in silico secretory proteins and potential effector proteins. Finally, 808 proteins from head and 267 proteins from salivary gland were identified to be potential effector protein encoding genes. Red boxes are the software’s used, corresponding to each filtering step. Taxonomic distribution and functional annotation was performed using six different databases (Nr, UniProt, Pfam, KO, GO, and COG). Numbers shown here are predicted peptides remaining after each step in the pipeline

**Fig. 3:**
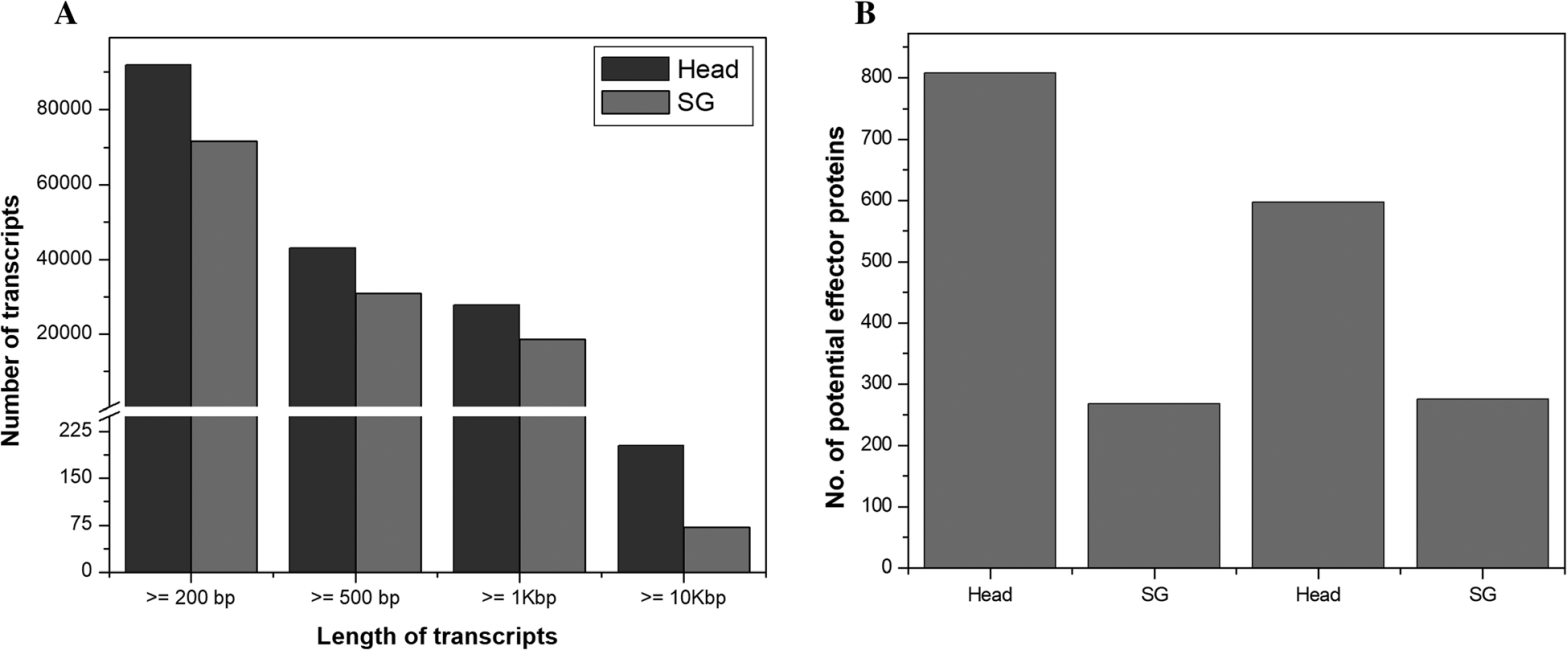
**a** Length distribution of transcripts after de novo assembly of *Spodoptera litura* head and salivary gland (SG) transcriptome using Trinity. **b** The number of potential effector proteins predicted in the head and salivary gland of *Spodoptera litura* using de novo based assembly and reference based assembly

*S. litura* has three kinds of salivary glands (SG), i.e. labial salivary glands, ventral eversible glands and mandibular glands (Fig. 1) [23–25]. The cDNA libraries from *S. litura* salivary glands (labial salivary gland and ventral eversible gland) and head (treated as a proxy of the mandibular gland) were sequenced using Illumina sequencing platform resulting in a total of 48.16 Mbp and 44.53 Mbp raw reads respectively. The workflow used for the processing of raw data is shown in Fig. 2. After removing adapters, ambiguous nucleotides and low-quality sequences with FASTQ quality filter tool (FASTQC), 41.68 Mbp (93.6%) reads from the head and 44.05 Mbp (91.47%) reads from the SG were retained. Subsequently, these clean reads were assembled using the de novo assembling program Trinity, which generated 91,927 and 71,706 transcripts from the head and SG, respectively (Table 1). An average of 97.25% of the processed reads were aligned to the assembled transcripts. In the head, the maximum length of a transcript was 33.54 Kbp, and the minimum was 201 bp. Similarly, it was 29.17 Kbp and 201 bp for SG. Based on all the transcripts generated from head and SG, the average transcript length was 1106 bp and 957 bp, respectively. In the head and salivary gland, the length of transcripts varied from ≥200 bp to ≥10 Kbp, with maximum transcript of ≥200 bp (Fig. 3a). The assembled transcripts were used for in silico protein prediction by TransDecoder. In total, 31,450 proteins from the head and 22,486 proteins from the salivary gland were predicted. Among these predicted proteins, 18,754 head proteins and 11,992 salivary gland proteins were full-length proteins (Table 1).

**Table 1:** Summary of transcriptome data from *Spodoptera litura* head and salivary gland

Statistics	Head	SG
Raw reads (in million)	44.53	48.16
Processed reads (in million)	41.68	44.05
Percentage of high-quality reads (%)	93.6	91.47
Transcripts generated	91,927	71,706
Average transcript length (bp)	1106	957
Number of predicted proteins	31,450	22,486
Number of complete proteins	18,754	11,992
Number of secretory proteins	899	330
Number of potential effector proteins	808	267

### In silico prediction of secretory proteins

We utilized a previously reported in silico pipeline to predict the secretome/secreted proteins from the assembled transcriptomes [26]. First, we identified proteins with signal peptide using SignalP, and the proteins with D-score = Y1 were carried forward for the next step of the analysis. These proteins were then scanned for transmembrane spanning regions using TMHMM and proteins without transmembrane domains were retained. Finally, we used TargetP and WolfPSort to predict the subcellular localization. Proteins with location predicted as Loc = S^2^ using TargetP and proteins predicted as extracellular (Ext > 17) using WolfPSort, were retained as final secretory proteins dataset (in silico secretome) (Fig. 2). After these filtering steps, 899 and 330 proteins were identified as secretory in head and salivary gland, respectively (Table 1).

An additional filtering step was applied as the pathogenic effectors are fast-evolving and known to occur in expanded gene families [27]. We clustered all the secretory proteins using TRIBE-MCL [28] depending upon the family size, i.e. the number of proteins in an individual family. From the head, a total of 251 families were identified (ranging from 2 to 92 members, 89 singletons). From the salivary gland 127 families were identified (ranging from 2 to 19 members, 63 singletons). Protein families having a single member (singletons) were excluded. (Table 2). Eight hundred eight and two hundred sixty-seven proteins from the head and salivary gland respectively were predicted as potential effectors from the *de-novo* transcriptome data (Table 1, Additional file 2: Table S1). Meanwhile, from the published *S. litura* genome [3] we also did an independent reference based transcriptome assembly of head and SG (salivary gland). From this assembly, 595 and 271 potential effector proteins were predicted in head and SG (Additional file 3: Table S2). Potential effectors in head decreased in reference based assembly compared to de novo transcriptome assembly, while in SG they remained mostly similar (Fig. 3b). We have used the *de-novo* transcriptome data for all further analysis.

**Table 2:** Protein family prediction using MCL clustering

	Groups	Family size (number of proteins)	Frequency (number of families)
**Head**	Group 1	92	1
	Group 2	61	1
	Group 3	28	1
	Group 4	23	1
	Group 5	16	3
	Group 6	11 ≤ 15	5
	Group 7	6 ≤ 10	17
	Group 8	2 ≤ 5	130
**SG**	Group 1	19	1
	Group 2	16	1
	Group 3	12	2
	Group 4	6 ≤ 10	6
	Group 5	2 ≤ 5	54

### Functional annotation of potential effector proteins using public databases

**Fig. 4:**
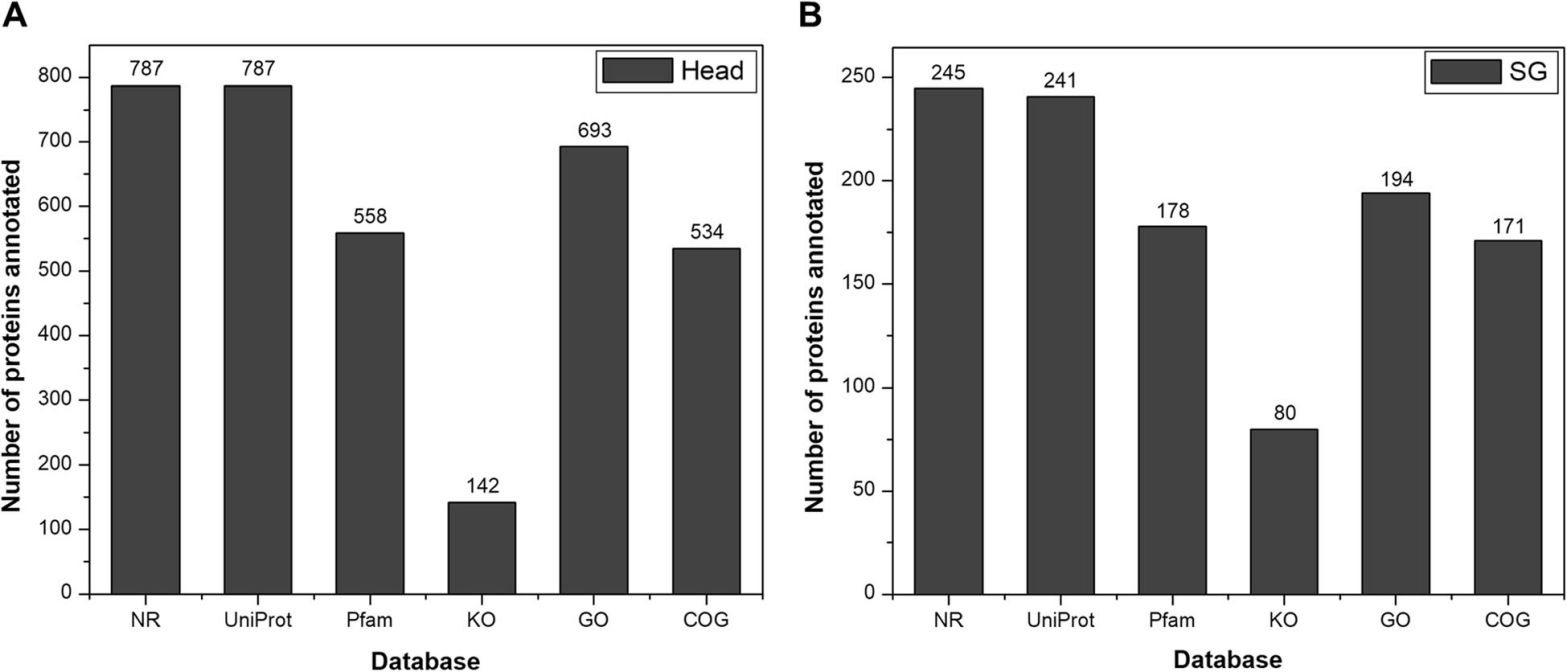
Potential effector proteins annotated with six public databases. In total, 808 proteins from the head and 267 proteins from the salivary gland were predicted to be potential effector proteins. These set of proteins from both the samples were simultaneously annotated with six different databases; NR (RefSeq non-reductant NCBI) and UniProt for taxonomic distribution and Pfam, KO (KEGG Orthology), GO (Gene Ontology) and COG (Clusters of Orthologous Groups) for functional annotation. Values at the top of each bar show the number of proteins annotated to corresponding database. Annotation of head effector proteins (**a**) and SG effector proteins (**b**)

**Fig. 5:**
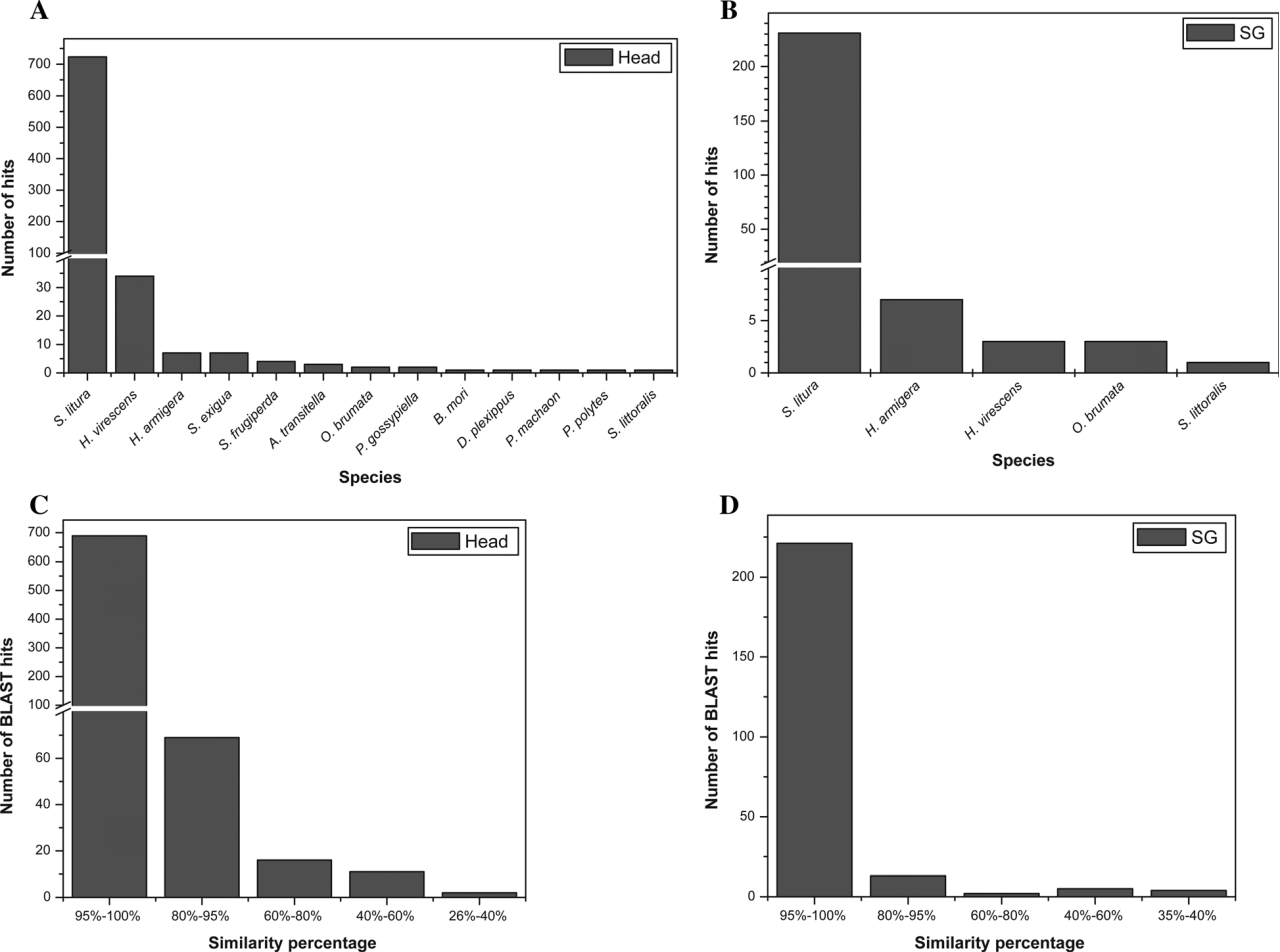
Phylogenetic Relationship and similarity percentage of all the potential effector proteins in the head and salivary gland. Similarity search of the proteins was carried out using BLASTp for species and similarity distribution. BLASTp was performed against all the sequences from the RefSeq non-redundant NCBI database (Order: Lepidoptera) at an e-value cut-off of 1e-5. Fig. A and B are for species distribution of the BLAST result for each protein in the head and salivary gland, respectively. Whereas Figs. C and D are, similarity percentage distribution of BLAST hits for each protein

Sequence similarity search of potential effector proteins were performed for functional annotation and taxonomic profiling. In Fig. 4, the number of proteins annotated to each public database in the head (Fig. 4a) and the salivary gland is shown (Fig. 4b). Annotation summary of all the potential effector proteins in this study is provided in Additional file 2: Table S1. Protein sequences were compared to the Lepidopteran database from Nr-NCBI and UniProt to know the phylogenetic relationship amongst the taxonomic class Insecta. The results against Nr-NCBI database showed that 787 out of 808 proteins from the head and 245 out of 267 proteins from the salivary glands were annotated. When analyzing the species distribution, the highest percentage of the sequences matched to *Spodoptera litura* (91.86%) [3] and *Heliothis virescens* (4.32%) for head proteins (Fig. 5a). In salivary gland proteins, the highest percentage of the sequences matched to *S.litura* (94.28%) [3] and *Helicoverpa armigera* (2.85%) (Fig. 5b). Also, the results against UniProt databases showed that 787 out of 808 proteins from the head and 241 out of 267 proteins from the salivary gland were annotated. Since the UniProt database did not have fully sequenced *S.litura* genome sequences deposited, most of the proteins were annotated to *S. frugiperda* (Additional file 1: Figure S1). The E-value and sequence similarity distribution of the top BLAST hits for each protein added strength to the analysis and general quality of the predicted proteins. The similarity distribution of potential effector proteins that have significant BLASTp hits against the Nr-NCBI protein database is shown in Fig. 5c and Fig. 5d.

**Fig. 6:**
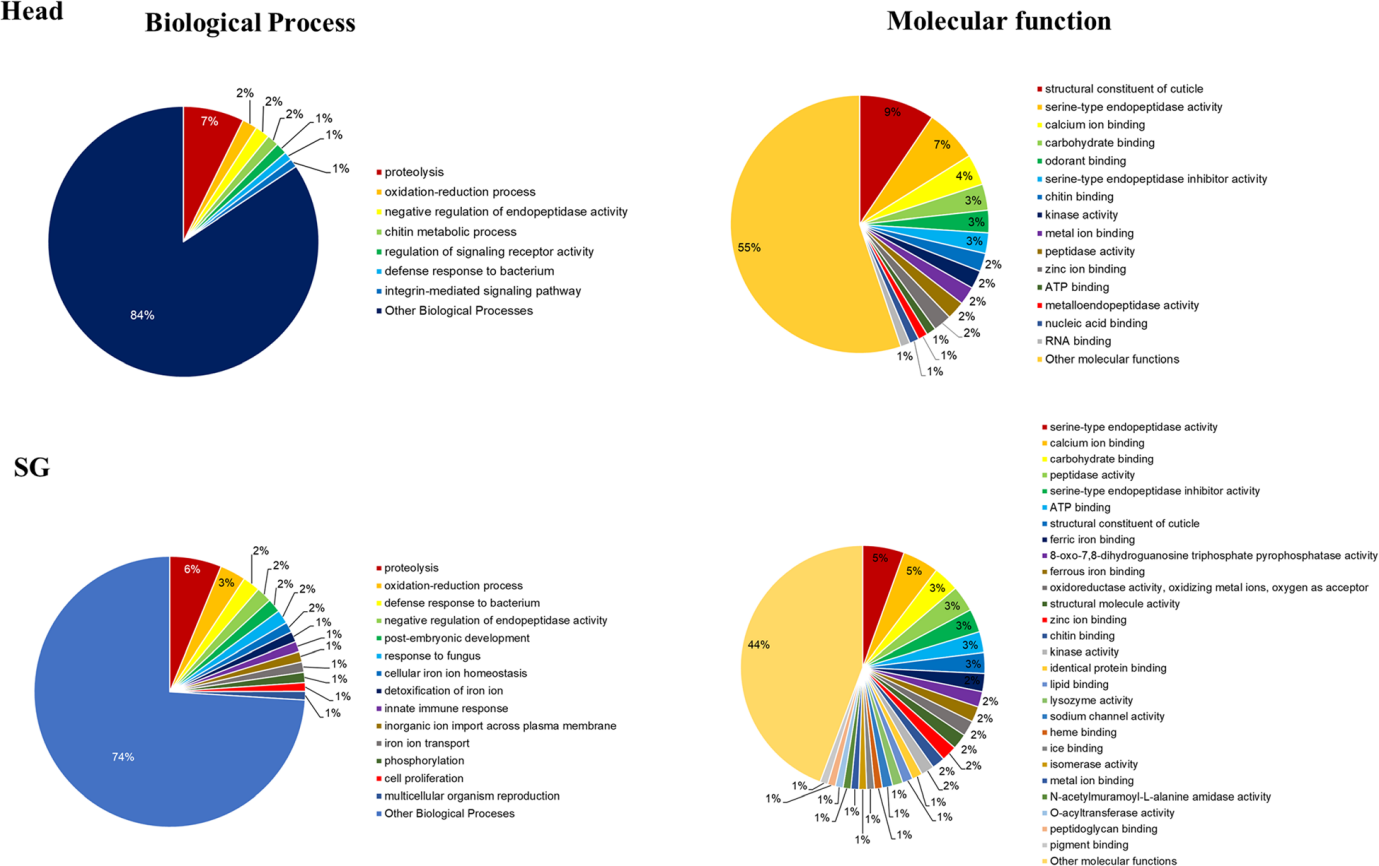
The enriched Gene Ontology categories (biological process and molecular function) for all predicted potential effector proteins. In total, 693 of 808 proteins in the head and 194 of 267 in salivary gland were assigned GO term. GO categories were assigned to proteins using PANNZER-2. GO terms with ≥1% abundance in annotated proteins are shown here. GO terms with < 1% abundance are grouped into “other biological processes” and “Other molecular function”

Gene Ontology (GO) assignments were used to functionally classify the predicted proteins from *S. litura* head and SG transcriptome using PANNZER2 web server. GO terms were assigned to 693 (85.77%) and 194 (72.66%) potential effector proteins in the head and SG, respectively. GO consists of three ontologies: ‘biological process’,‘molecular function’, and ‘cellular component’. Differential distribution of the protein functions between the head and SG (more than 1% abundance) are depicted in Fig. 6. The top five annotated categories of each GO ontology are shown in Additional file 1: Figure S2A. In the head, the three most abundant categories of ‘biological process’ are ‘proteolysis’(7.27%),‘oxidation-reduction process’(1.80%) and ‘negative regulation of endopeptidase activity’(1.73%). In the ‘molecular function’, proteins were mainly distributed amongst ‘structural constituent of cuticle’(9.44%) and ‘serine-type endopeptidase activity’ (6.66%) (Fig. 6, Additional file 1: Figure S2B). Whereas, in the salivary gland, the two most abundant categories in the ‘biological process’ are ‘proteolysis’ (6.18%) and ‘oxidation-reduction process’ (3.09%). In the ‘molecular function’, proteins were mainly distributed amongst ‘serine-type endopeptidase activity’ (5.49%) and ‘calcium ion binding’ (4.81%) (Fig. 6, Additional file 1: Figure S2B). GO terms were also assigned to potential effector proteins, predicted by reference based transcriptome assembly [3]. In the Pie Chart most of the categories were repeated at similar hierarchical positon, such as proteolysis in the head as well as in the SG (Additional File 1: Figure S3A &B). However, few categories were either missing or new were assigned.

EuKaryotic Orthologous Groups (KOG) is a version of the Clusters of Orthologous Groups (COG) for identifying orthologous and paralogous proteins in eukaryotic organisms [29]. In total, 534 (66.09%) proteins from the head and 171 (64.04%) proteins from the salivary gland were categorized into 16 different categories of the COG database. In the head, the group ‘function unknown’ had the most number of proteins (43.32%) followed by ‘post-translational modification, protein turnover’. In the salivary gland similar pattern was observed, where the group ‘function unknown’ had the most proteins (37.89%), followed by ‘post-translational modification, protein turnover, and chaperones’ (15.78%) (Additional file 1: Figure S4). The results from COG annotation suggested that many proteins predicted as effectors have unknown functions.

**Fig. 7:**
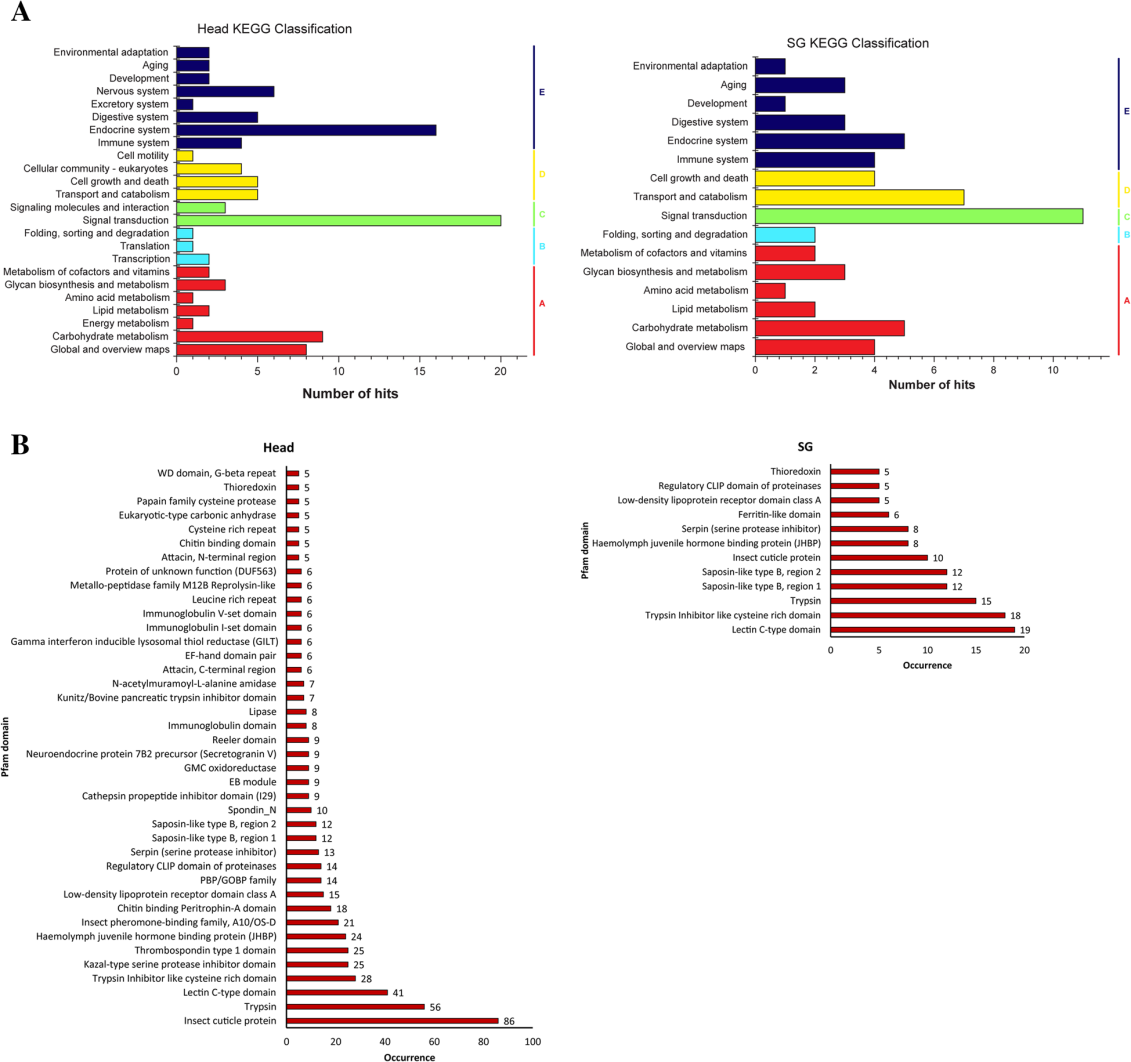
**a** The predicted KEGG pathways for all potential effector proteins. In KO database, a small number of proteins were annotated i.e. 142 out of 808 in the head and 80 out of 267 in the salivary gland were annotated. The proteins in the second hierarchy of the KEGG pathway were assigned to 5 categories A: Metabolism, B: Genetic Information Processing, C: Environmental Information Processing, D: Cellular Processes, E: Organismal Systems. **b** Predicted Pfam domains in the transcriptome of the head and Salivary gland of *S. litura.* In Pfam database, 558 out of 808 proteins in the head and 178 out of 267 proteins in the salivary gland were annotated. Pfam domains were mapped on potential effector proteins via Pfam standalone software using the default parameters. Numerical values at the top of each bar represent the number of times that domain has occurred in the annotated proteins. The domains which have occurred ≥5 times in the annotated proteins are shown here

All of the sequences were further assigned to the reference canonical pathways in the Kyoto Encyclopaedia of Genes and Genomes (KEGG), to study which biological pathways and molecular interactions are active in the salivary glands [30]. Only a small percentage of proteins were mapped to the KEGG pathway database. KEGG mapping revealed the different distribution of biological pathways for head and salivary glands (Fig. 7a). A total of 142 (17.57%) proteins from the head and 80 (29.96%) from the salivary gland were mapped to 106 and 58 pathways (Additional file 4: Table S3). The salivary glands act as a specific organ for the salivary macromolecule production and, as a result, have a high level of metabolic activity. Such a characteristic feature was shown in a study [31] by detecting the dense cytoplasm and organized whorls of rough endoplasmic reticulum in the salivary-gland cells. Consequently, “metabolism” is important among these pathways in the salivary glands (26 head proteins and 17 salivary gland proteins) (Fig. 7a). The KEGG Orthology (KO) classification shows that the salivary glands might be active in metabolism, transport and binding.

From the potential effector proteins, we wanted to identify the motifs/domains present. A Pfam database assigned domains to 558 (69.06%) head proteins and 178 (66.67%) salivary gland proteins, and the rest were unknown proteins. This search identified 168 unique domains in the head sample and 107 unique domains in the SG sample (Additional file 5: Table S4). The Pfam domains with occurrence greater than or equal to 5 are shown in Fig. 7b. The top three most abundant domains in the head sample are ‘Insect cuticle protein’, ‘Trypsin’ and ‘Lectin C-type domain’. In the salivary gland, the top three most abundant domains are ‘Lectin C-type domain’, ‘Trypsin Inhibitor like cysteine rich domain’ and ‘Trypsin’. ‘Ferritin-like domain’, ‘Regulatory CLIP domain of proteinases’, ‘Haemolymph juvenile hormone binding protein’, ‘EF-hand domain’, ‘Serpin’, ‘Lipase’, ‘GMC oxidoreductase’ are some of the other interesting domains of predicted effector proteins (Additional file 6: Table S5). Pfam domains were predicted for individual protein families that were clustered using MCL analysis, and selected domains are shown in Table 3. Interestingly, there are proteins with no Pfam domain but are functionally annotated to the Nr database, which shows the characteristic of effector-like proteins (Additional file 7: Table S6). Proteins annotated to both Nr and Pfam databases, have digestive and hydrolyzing properties such as serine proteases, serpin, glycosyl hydrolases, lipases, phospholipases, cathepsin, trypsin, metalloproteases, endochitinase and GMC oxidoreductase. We also identified some potential effector proteins such as calumenin (with calcium ion binding properties), chemosensory proteins (CSP) and odorant binding proteins (OBP), which are utilized by the insects to locate host plants and predators.

**Table 3:** The *S. litura* transcriptome protein families according to MCL analysis with their predicted Pfam domains

	Family size (number of proteins)	Frequency (number of families)	Major predicted Pfam domains
**Head**	92	1	Insect cuticle protein
	61	1	Regulatory CLIP domain of proteinases//Trypsin
	28	1	Haemolymph juvenile hormone binding protein (JHBP)
	23	1	Insect pheromone-binding family
	16	3	Lectin C-type domain, PBP/GOBP family, Reeler domain//Spondin_N
	14	2	Serpin (serine protease inhibitor), Trypsin Inhibitor like cysteine rich domain
	12	1	Lectin C-type domain
	11	2	Immunoglobulin domain
	10	2	Protein of unknown function (DUF563), Leucine rich repeat
	9	1	Metallo-peptidase family M12B Reprolysin-like
	8	4	Attacin, Kazal-type serine protease inhibitor domain, Lipase
	7	3	N-acetylmuramoyl-L-alanine amidase, Chitin binding Peritrophin-A domain
	6	7	Gamma interferon inducible lysosomal thiol reductase (GILT), GMC oxidoreductase, Neuroendocrine protein
	5	13	Cysteine-rich secretory protein family, Chitin binding domain, Cathepsin propeptide inhibitor domain
	4	23	EF-hand domain pair, EB module, Lectin C-type domain, Sulfotransferase family
	3	25	Ferritin-like domain, Carboxylesterase family, Aldo/keto reductase family, Copper/zinc superoxide dismutase
	2	69	Sulfatase, PBP/GOBP family, Thioredoxin
**SG**	19	1	Insect cuticle protein
	16	1	Trypsin
	12	2	Lectin C-type domain//Lectin C-type domain, sulfatase
	10	1	Haemolymph juvenile hormone binding protein (JHBP)
	9	2	Ferritin-like domain, Trypsin Inhibitor like cysteine rich domain
	8	1	Serpin (serine protease inhibitor)
	6	2	EF-hand domain pair, Immunoglobulin domain
	5	6	Cathepsin propeptide inhibitor domain (I29), Chitin binding Peritrophin-A domain
	4	10	WIF domain//EGF-like domain, Destabilase, Chitin binding domain, Gamma interferon inducible lysosomal thiol reductase (GILT)
	3	14	Thioredoxin-like domain, Carboxylesterase family, ML domain, Sema domain
	2	24	Lipase, Attacin, Aldo/keto reductase family, Sulfotransferase family, Peptidase S24

**Fig. 8:**
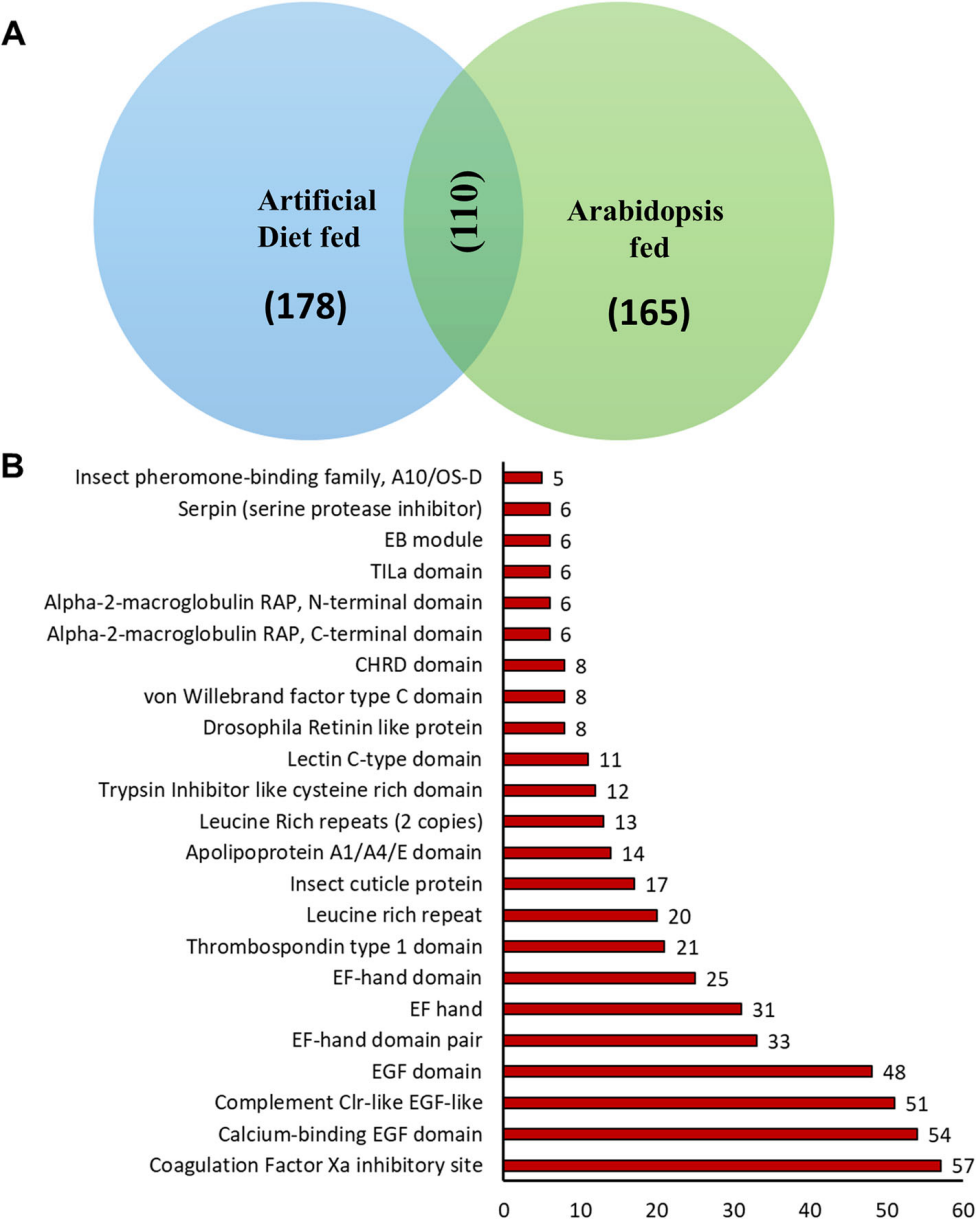
**a** Potential effector proteins from salivary glands present in two different environment conditions i.e. artificial diet fed and Arabidopsis fed. Venn diagram depicting the number of potential effector proteins in artificial diet fed and Arabidopsis fed. 110 potential effector proteins are common under both conditions. **b** Predicted Pfam domains in the common effector proteins*.* In total, there are 110 common proteins between artificial diet fed and Arabidopsis fed salivary gland. Pfam domains were assigned to these proteins through Pfam standalone software using the default parameters. Numerical values at the top of each bar represent the number of times that domain has occurred in the common proteins. The domains that occurred ≥5 times in the common proteins are shown here

We also performed *S. litura* reference based assembly and effector prediction in SG, upon interaction with the host plant. The insects were also allowed to feed on *Arabidopsis thaliana* before gland isolation. A total of 288 potential effector proteins were predicted in SG in fed condition (Additional file 8: Table S7). Out of the total, 165 proteins were found specific to host-plant interaction and 110 proteins were common between fed and unfed conditions (Fig. 8a). Pfam domains were assigned to 110 common proteins (Fig. 8b) and to 165 specific proteins (Additional file 1: Figure S5).

### Gene expression analysis in different tissues of *S. litura* by RT-PCR

**Fig. 9:**
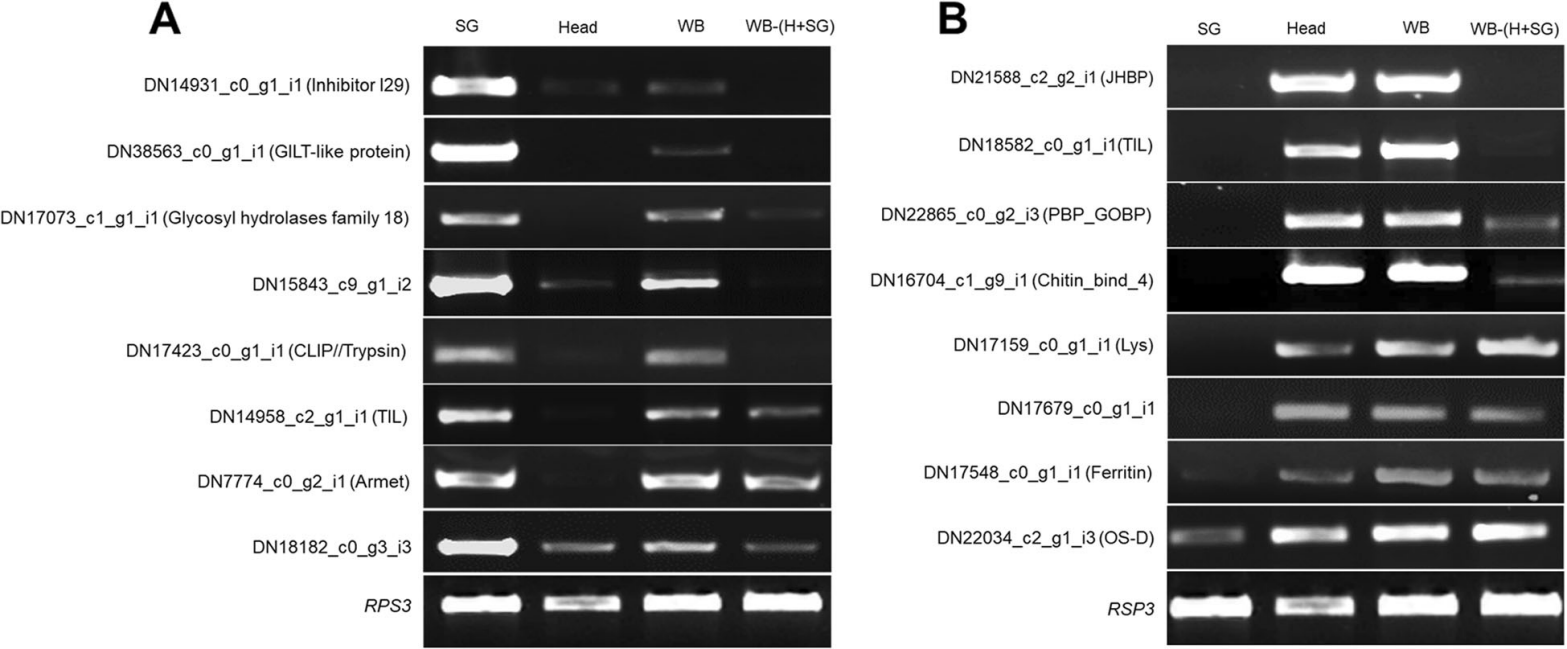
RT-PCR amplification of most highly expressed potential effector encoding genes in (**a**) salivary gland (**b**) head. The expression is checked in different tissues, SG (salivary gland), WB (whole body of insect), WB-(H + SG) (whole body without head and salivary gland)

Predicted potential effector encoding genes were examined for their expression and specificity in different insect tissues, i.e. salivary gland (SG), head (H), whole body (WB) and whole body excluded of head and salivary gland WB-(H + S). Eight genes from the SG and head each, were selected for amplification based on their Pfam domain and high expression (RPKM). Out of 8 selected genes from SG transcriptome, RT-PCR results (Fig. 9a) showed that the first five genes (DN14931_c0_g1, DN38563_c0_g1_i1, DN17073_c1_g1_i, DN15843_c9_g1_i2, DN17423_c0_g1_i1) were only expressed in SG and WB, indicating that the expression is specific to the salivary gland. Two genes (DN14958_c2_g1_i1, DN7774_c0_g2_i1) were expressed in WB-(H + SG), SG and WB, indicating its uniform expression in insect, except head. DN18182_c0_g3_i3 was expressed in all the four selected tissues of the insect, but its expression level was high in SG. All the selected genes thus validated transcriptome data.

Expression analysis in the head (a proxy for mandibular glands) (Fig. 9b) exhibits that the two genes (DN21588_c2_g2_i1, DN18582_c0_g1_i1) were specific to head. Five genes (DN22865_c0_g2_i3, DN16704_c1_g9_i1, DN17159_c0_g1_i1, DN17679_c0_g1_i1, DN17548_c0_g1_i1) were expressed in head, WB and WB-(H + SG) indicating their uniform expression in all the tissues except SG (which has ventral eversible and labial salivary gland). Hence, these genes could have additional functions apart from being potential effector proteins. DN22034_c2_g1_i3 indicates expression in all the tissues of the insect. However, the level of expression was less in SG compared to the head and other selected tissues. In a nutshell, all the selected genes predicted by the effector prediction pipeline denoted the specific expression in head and SG.

## Discussion

The common cutworm, *S. litura* (Fab.) is a major generalist agricultural pest worldwide. However, little is known about the mechanisms used by Spodoptera for a successful invasion of a large set of host plants. Effectors have evolved to bind and modify the host molecules and perturb plant processes [32, 33]. The enzymatic activities of effectors are diverse and include protease, hydrolase, phosphatase, kinase, transferase, and ubiquitin ligase activities. Some effectors do not carry enzymatic activities and act by binding to the host proteins to modulate their functions [34, 35]. The genes encoding effector proteins are commonly identified in multigenic clusters created by gene duplication and diversification, with the conserved intergenic regions and highly diversified coding regions. This unusual conservation is a unique feature of effectors, suggesting the rapid evolution in response to selection pressures [36]. Many of the predicted effector proteins are required by the insects to digest and assimilate plant material during feeding [17, 18, 20]. A large expansion of the gustatory receptor (GR), cytochrome P450 (P450), carboxylesterase (COE) and glutathione-*S*-transferase (GST) gene families have been reported in *S*. *litura* which are responsible for its wide host range [3]. We briefly describe the key potential effector proteins predicted from our transcriptome data with *S. litura* (Fab.) and their known functions:

### Detoxifying enzymes and digestive inhibitors

Plants limit herbivore feeding through constitutive and inducible defenses. The inducible defense arsenal includes secondary metabolites like glucosinolates, alkaloids, terpenes, lectins, and proteinase inhibitors [37–41]. To overcome these barriers, insects have evolved detoxifying enzymes such as glutathione S-transferases and esterases. We also found esterases in our transcriptome (SG; DN8177_c0_g1_i1 and head: DN13269_c0_g1_i1). Detoxifying enzymes are found in salivary glands of aphids and other insects like potato leafhopper, *Empoasca fabae* [12, 13, 21]*, N. lugens* [13]*,* rice green planthopper (*Nephotettix cincticeps*) [14]. We found many enzyme inhibitors in *S. litura* secretome, like kazal-type serine protease inhibitors (head; DN19384_c0_g2_i2), metalloproteases (SG; DN14958_c2_g1_i1 and head; DN18582_c0_g1_i1) and serine proteinase inhibitors (SG; DN11199_c0_g1_i1). Previously they were also identified in the salivary gland of *Helicoverpa armigera* [42] pea aphid (*Acyrthosiphon pisum*) [43]*,* vetch aphid (*Megoura viciae*) *and M. persicae* [44]*.* Although the function and mode of action of these proteases in plant-herbivore interactions are not well studied, they are predicted to be involved in detoxifying defense proteins of plants apart from its possible role as effectors.

### Glucose oxidase

Glucose oxidase (GOX) is present in salivary secretions of many insect pests and leads to suppression of plant defense. *Helicoverpa zea* secretes GOX in *Nicotiana tabacum,* resulting in suppression of nicotine production and jasmonic acid signaling [20]. GOX from *H. zea* and *Spodoptera exigua* elicits plant defense in *Solanum lycopersicum and Zea mays* [45]*.* It is predicted as an effector in grain aphid, *Sitobion avenae* [44]. Our study also found a member of the same family (head; DN20219_c0_g1_i2) in *S. litura* secretome*.*

### Chemosensory and odorant binding proteins

Odorant-binding proteins (OBPs) (SG; DN13422_c0_g1_i1 and head; DN15662_c0_g1_i1, DN15765_c0_g1_i1, DN15768_c0_g1_i1) and chemosensory proteins (CSPs) (SG; DN15038_c0_g1_i1, DN20500_c2_g1_i1 and head; DN19969_c0_g1_i1, DN19997_c0_g2_i3) were found in secretome of *S. litura*. Previous studies revealed OBPs and CSPs in the salivary gland transcriptome of *N. lugens* [13] and *M. persicae* [19]*.* Insect OBPs and CBPs are expressed in antennae and mouthparts, and involved in semiochemical communication [10]. OBPs are found in the salivary gland of mosquito, *Anopheles gambiae* and is predicted to manipulate the host physiology by scavenging host amines [46]. CSPs are known as abundant proteins in the mandibular gland of painted lady butterfly (*Vanessa cardui*) and New Zealand red admiral butterfly (*Vanessa gonerilla*) [47] and play an important role in host plant recognition. Chemosensory protein Mp10 of the green peach aphid *Myzus persicae* is an effector that suppresses plant reactive oxygen species (ROS) bursts in Arabidopsis and is evolutionarily conserved [10].

### Calcium (Ca^2+^) ion binding proteins

Plant Ca^2+^ signaling is crucial for the recognition of herbivory and activation of downstream defense pathways [21, 48]. Studies have revealed that the effectors downregulate Ca^2+^ elevation [21, 49]. Ca^2+^ binding protein, calumenin (SG; DN14065_c0_g1_i1, head; DN23273_c0_g1_i2) calreticulin (SG; DN17937_c0_g1_i1), armet (head; DN8860_c0_g2_i1, SG; DN7774_c0_g2_i1), and 8 Ca^2+^ binding EF-hand domain containing proteins (head; DN12467_c0_g3_i1, DN14059_c0_g1_i1, DN15442_c0_g2_i1, DN15442_c0_g2_i2, DN18248_c0_g1_i1, DN18248_c0_g1_i2 and SG; DN20185_c0_g1_i2, DN20185_c0_g1_i3, DN5404_c0_g1_i1) were present in *S. litura* secretome. These proteins can bind to intracellular Ca^2+^ ion of the host plant and suppress plant defense [10, 16]. For instance, in *Vicia faba,* salivary secretion of aphid releases Ca^2+^ binding proteins, which sequester Ca^2+^ and leads to the contraction of forisomes. Such contraction avoids the plugging of sieve elements and allows aphid to continuously feed on the phloem of the host plant [43]. Similarly, knockdown of salivary Ca^2+^ binding protein *NlSEF1* in *N. lugens* decreases fecundity and increases H_2_O_2_ and mortality of larvae while feeding on rice [16]. The Ca^2+^ binding calumenin, calreticulin and EF-hand domain proteins are also reported as effectors in the salivary transcriptome of *Sitobion avenae* [15] and *A. pisum* [9].

### Clip domain serine proteinases

Through our study, clip domain serine proteinases (clip-SPs) (SG; DN17423_c0_g1_i1, head; DN15833_c0_g1_i1) were found in *S. litura’s* salivary secretion. Clip-SPs are an extracellular and non-digestive enzyme, which are known to be involved in embryonic development and the defense responses [50, 51]. Apart from their known roles in embryonic development and defense, they may have a role as effector proteins.

### Lipases

Our studies, predicted the presence of lipases (SG; DN10494_c0_g1_i1, and head; DN21574_c0_g2_i2, DN19769_c0_g1_i1, DN21661_c0_g1_i1) in *S. litura* salivary secretion. They were reported in the salivary glands of different insects, including *Sitobion avenae* [15]*, Empoasca fabae* [12]*,* seed feeding bug, *Oncopeltus fasciatus*, [52] *Mayetiola destructor* [53] and tobacco hornworms, *Manduca sexta* [54] and involved in oral digestion. The role of lipases as potential effectors are known in *Fusarium graminearum* [55]. Application of lipases from grasshopper, *Schistocerca gregaria* to wounded leaves of Arabidopsis elicited the rapid accumulation of oxylipins such as OPDA, and jasmonic acid [56]. Some Phospholipases (head; DN19769_c0_g1_i1, DN21661_c0_g1_i1) have also been found in our transcriptome data. Phospholipases in *S. avenue* [15] hydrolyze the plant phospholipids, that play a crucial role in lipid synthesis and lipid-derived signaling cascades [44, 57].. Thus lipase and phospholipases secreted from *S. litura* could affect the plasma membrane composition and integrity.

### Peroxidases

Reactive oxygen species (ROS) play an important role in plant defense against various insects. It activates the early signaling in Arabidopsis after cabbage aphid, *Brevicoryne brassicae* infestation [58]. ROS is toxic to herbivores, so they release peroxidases to supress the ROS production, thus acting as effectors. Peroxidases (SG: DN19481_c1_g1_i9, head: DN12032_c0_g1_i1) which are oxidoreductase enzymes were found in our analysis. Peroxidase was previously reported in the salivary gland of *M. destructor* [11]*, A. pisum* [9], *M. viciae* [44]*,* and *S. avenae* [15].

## Conclusion

In conclusion, our study reveals the identities of effector encoding genes in *Spodoptera litura* salivary glands. Effector proteins secreted during feeding, play an essential role in *S. litura*-host interaction. Detoxifying enzymes (esterases, kazal-type serine protease inhibitors, metalloproteases and serine proteinase inhibitors), glucose oxidase, chemosensory proteins (odorant binding proteins and chemosensory proteins), calcium ion binding proteins (calumenin, calreticulin, and armet), clip domain serine proteinases, lipases, phospholipases and peroxidases are the major potential effector protein families identified in our study. Functional and mechanistic characterization of these effector proteins will provide deeper insight into the insect-mediated suppression of plant defense.

## Methods

### *Spodoptera litura* growth and salivary gland isolation

An inbred strain of *Spodoptera litura* (Fabricius strain) was used in the study. Eggs were obtained from NBAIR (Bangalore, India) live insect repository (accession no. is NBAII–MP-NOC-02). After hatching, larvae (50) were reared on gram flour-based artificial diet at 25 ± 2 °C with 8 h /16 h of the light-dark period [59]. For Arabidopsis fed *S. litura,* the insects (50) were allowed to feed on 5–6 week old Arabidopsis plants for 24 h prior to gland isolation. *S. litura* 5th instar caterpillars were cold anesthetized (− 20 °C for 20 min) on a cold petri dish containing 1X phosphate buffer saline (PBS) solution. An incision between head and thorax was made for labial salivary glands (LSG) [49] and ventral eversible gland (VEG) [24], glands were pulled out using sterilized forceps. In total, 50 pairs of LSG, VEG and 50 mandibular glands (whole head is used as a proxy for extremely small mandibular glands) were isolated for further experiments (Fig. 1).

### RNA isolation, library construction, Illumina sequencing, and de novo assembly

RNA was isolated from three samples, i.e., salivary gland (SG) (pool of 50 pairs of LSG and VEG) of both artificial diet, plant (*Arabidopis thaliana*) fed insects and head (pool of 50) using the Qiagen RNeasy mini kit. Total RNA was extracted, quantified and quality was assessed by Bioanalyzer (Agilent technologies). RNA sequencing libraries were prepared with Illumina-compatible NEBNext® Ultra™ directional RNA library prep kit (New England Biolabs, MA, USA) following the manufacturer’s instruction. In brief, RNA was taken for mRNA isolation, fragmentation, and priming. Fragmented and primed mRNAs were further subjected to first-strand cDNA synthesis followed by second-strand synthesis. Then the double-stranded cDNAs were purified using HighPrep magnetic beads (Magbio Genomics Inc., USA). Purified cDNAs were then end-repaired, adenylated and ligated to Illumina multiplex barcode adapters. Followed by this, PCR and purification was done. Continued with library preparation and quantification, its fragment size distribution was analyzed and sequenced, which generated 150*2 bp paired-end reads. The median insert size for the library is 180 bp to 580 bp for both the samples.

We applied effector-mining strategy as described in [10] to generate a list of *S. litura* candidate-effector proteins. Before assembly, raw data quality check was done using FastQC (https://www.bioinformatics.babraham.ac.uk/projects/fastqc/), followed by pre-processing using Cutadapt, which includes the removal of adapter sequences and low-quality bases (Q score > 30). All the high quality-filtered reads were assembled using the *de Bruijn* graph-based tool Trinity [60] with default k-mers, i.e., 25, which represents a novel method for the efficient and robust de novo reconstruction of transcriptomes from RNA-Seq data. The relative abundance of each transcript was reported as Reads Per Kilo-base of transcript, per Million mapped reads (RPKM). These assembled transcripts were used for the prediction of open reading frames (ORFs) using TransDecoder (https://github.com/TransDecoder/TransDecoder/releases), which identifies candidate coding regions within transcript sequences.

### Reference-based transcriptome analysis of *Spodoptera litura* salivary gland

The raw FASTQ file from Illumina sequencing was used for the removal of adapter sequences and low-quality bases using TrimGalore (https://github.com/FelixKrueger/TrimGalore). All the clean reads after adapter removal were used for further reference-based assembly. We mapped all the clean reads from the different tissues (Head and Salivary Gland) to the *S. litura* reference genome (ASM270686v2) [3] using HISAT2, a fast and sensitive alignment program for mapping next-generation sequencing reads [61]. The StringTie program, a fast and highly efficient assembler of RNA-Seq alignments into potential transcripts was used to assemble the transcriptomes and estimate the transcript abundance [62]. The GTF output file from StringTie was used in the TransDecoder program to identify the ORFs within the transcripts. The predicted coding regions were then further used for predicting the candidate effector proteins.

### Identification of in silico secretory proteins and potential effector proteins

The predicted proteins were used for identification of secretory proteins from *S. litura* transcriptome. The predicted proteins were filtered for the presence of signal peptide and the absence of transmembrane helices (Fig. 2). Signal peptides and cleavage sites were identified using SignalP 4.0 [63], and transmembrane domains were predicted using TMHMM v. 2.0 [64], subcellular localization was predicted using TargetP-2.0 [65] and WolfPSort [66]. Predicted proteins with a signal peptide, no transmembrane domains and complete proteins (i.e., with a start and stop codon) were considered to be secretory protein-encoding genes. Effectors are known to be fast-evolving [27]. Hence, they occur in expanded gene families, therefore resulting proteins were filtered and clustered into families based upon amino acid homology described by [67]. The Markov Cluster Algorithm (MCL) was used via TRIBE-MCL [28] using default parameters for the clustering of these proteins into families. Proteins families with two or more members were retained, and these proteins are reported to be potential effector protein-encoding genes (Fig. 2).

### Annotation of potential effector proteins

Potential effector proteins were annotated to six different public databases (Nr, UniProt, Pfam, KO, GO, and COG) for taxonomic profiling and functional annotation. Two databases were downloaded for order Lepidoptera (Taxonomy ID: 7088); one is containing 884,097 protein sequences from RefSeq non-redundant NCBI database and another containing 400,601 protein sequences from UniProt database. Homology search of the potential effector proteins was performed using BLASTp at an E-value cut-off of 1e-5 against the constructed database. Gene ontology (GO) analysis was performed to identify the putative function of the potential effector proteins. Protein ANNotation with Z-score (PANNZER) annotation server (http://ekhidna2.biocenter.helsinki.fi/sanspanz/) was used to perform GO analysis with a scientific name of query species: *S. litura*. Pfam domains were assigned to potential effector proteins via HMMER 3.1b2 (hmmscan) standalone software using the default parameters. Pathways were annotated using KEGG Automatic Annotation Server Ver. 2.1 (KAAS) to the manually curated Kyoto Encyclopedia of Genes and Genomes (KEGG) database. Gene data set was manually selected for all the 29 organisms under insects (3,94,729 sequences). Human disease-associated pathways were excluded from this analysis. Potential effector proteins were also mapped to Clusters of Orthologous Groups of proteins using EggNOG mapper (http://eggnogdb.embl.de/#/app/emapper) with the taxonomic scope set to Lepidoptera.

### Gene expression analysis in different tissues of *S. litura* by RT-PCR

To validate the expression of genes encoding potential effectors, we conducted a semi-quantitative RT-PCR in different tissues of insect; salivary glands (SG), head (H), whole body (WB), and whole body excluding head and salivary glands (WB-(H + SG)). Total RNA was extracted using the Qiagen RNeasy mini kit. DNAase (Turbo DNAse Ambion) treatment was done to eliminate DNA contamination. DNA-free total RNA (1 μg) was converted into single-stranded cDNA using a mix of oligo-dT20 primers with the Omniscript cDNA synthesis kit (Qiagen). For RT-PCR, gene-specific primers (Additional file 9: Table S8) were used, which amplified about 500 bp region of the gene. *RPS3* gene [68] was used as a reference for equal loading. RT- PCR conditions were used as - 5 min at 95 °C for initial denaturation, 30 s at 95 °C, annealing for 30 s at 57 °C, extension for 1 min at 72 °C (30 cycles) and final extension 10 min at 72 °C. Eight genes were selected for amplification based on their Pfam domain abundance and high expression (RPKM). The raw images of gel pictures corresponding to Fig. 9 are shown in Additional file 10: Figure R1-R17.

## Supplementary Information

**Additional file 1: Figure S1.** Species distribution and similarity percentage of all the potential effector proteins in head and salivary gland against UniProt (Order: Lepidoptera). **Figure S2.** Enriched GO terms. **Figure S3.** Enriched GO terms of potential effector proteins assembled by referenced genome of *S. litura.* **Figure S4.** The enriched COG functions for predicted potential effector proteins in head and salivary gland of *S. litura.* **Figure S5.** The predicted Pfam domains of potential effector proteins that only present in plant fed salivary gland (SGF) of *S. litura. (PDF 812 kb)*

**Additional file 2: Table S1.** Annotation summary of potential effector proteins.

**Additional file 3: Table S2.** Annotation summary of potential effector proteins (based of reference genome).

**Additional file 4: Table S3.** The list of KEGG pathways.

**Additional file 5: Table S4.** The Pfam domains found in the head and salivary gland transcriptome.

**Additional file 6: Table S5.** Proteins with interesting functions annotated to Pfam and Nr database.

**Additional file 7: Table S6.** List of proteins functionally annotated to Nr database but with no Pfam domain.

**Additional file 8: Table S7.** Annotation summary of potential effector proteins (based of Arabidopsis fed *S. litura*).

**Additional file 9: Table S8.** Primers used for RT-PCR of highly expressed genes in head and salivary gland.

**Additional file 10: Figure R1-R17.** Raw and unprocessed gel pictures correspondingto Fig. 9.

## Data Availability

The raw datasets generated during the sequencing of current study are available in the BioProject-NCBI repository under the accession PRJNA544612. *S. litura* reference genome (ASM270686v2) is used for reference based assembly.

## Abbreviations

PAMP: Pathogen-Associated Molecular Pattern
PRR: Pattern Recognition Receptor
PTI: PAMP-Triggered Immunity
ETI: Effector-Triggered Immunity
OS: Oral Secretion
HAMP: Herbivore Associated Molecular Pattern
SA: Salicylic Acid
NGS: Next-Generation Sequencing
PBS: Phosphate Buffer Saline
LSG: Labial Salivary Gland
VEG: Ventral Eversible Gland
SG: Salivary Gland
RPKM: Reads Per Kilobase of transcript, per Million mapped reads
ORF: Open Reading Frame
MCL: Markov Cluster Algorithm
GO: Gene Ontology
PANNZER: Protein ANNotation with Z-score annotation server
KEGG: Kyoto Encyclopedia of Genes and Genomes
KAAS: KEGG Automatic Annotation Server
COG: Clusters of Orthologous Groups
KOG: EuKaryotic Orthologous Groups
KO: KEGG Orthology
CSP: Chemosensory Proteins
OBP: Odorant Binding Proteins
GOX: Glucose Oxidase
Clip-SP: Clip domain Serine Proteinases
