# Supplementary material for: In silico identification of effector proteins from generalist herbivore Spodoptera litura
Source: BMC Genomics. 2020 Nov 23;21:819. doi: 10.1186/s12864-020-07196-4 (PMC7681983; doi:10.1186/s12864-020-07196-4)
Supplement: Supplementary file 1 — Additional file 1: Figure S1. Species distribution and similarity percentage of all the potential effector proteins in head and salivary gland against UniProt (Order: Lepidoptera). Figure S2. Enriched GO terms. Figure S3. Enriched GO terms of potential effector proteins assembled by referenced genome of S. litura. Figure S4. The enriched COG functions for predicted potential effector proteins in head and salivary gland of S. litura. Figure S5. The predicted Pfam domains of potential effector proteins that only present in plant fed salivary gland (SGF) of S. litura. (PDF 812 kb) [file 12864_2020_7196_MOESM1_ESM.pdf]

Identification of effector proteins from generalist herbivore, *Spodoptera litura* using *de novo* transcriptomics

Vinod Kumar Prajapati*, Mahendra Varma*, and Jyothilakshmi Vadassery

National Institute of Plant Genome Research (NIPGR), Aruna Asaf Ali Marg, New Delhi 110067, India

*equal contribution

*Corresponding author- Jyothilakshmi Vadassery: jyothi.v@nipgr.ac.in

Additional files

Additional file 1: Figure S1. Species distribution and similarity percentage of all the potential effector proteins in head and salivary gland against UniProt (Order: Lepidoptera).

Additional file 1: Figure S2. Enriched GO terms.

Additional file 1: Figure S3. Enriched GO terms of potential effector proteins assembled by referenced genome of *S. litura.*

Additional file 1: Figure S4. The enriched COG functions for predicted potential effector proteins in head and salivary gland of *S. litura.*

Additional file 1: Figure S5. The predicted Pfam domains of potential effector proteins that only present in plant fed salivary gland (SGF) of *S. litura.*

Additional file 2: Table S1. Annotation summary of potential effector proteins.

Additional file 3: Table S2. Annotation summary of potential effector proteins (based of reference genome)

Additional file 4: Table S3. The list of KEGG pathways.

Additional file 5: Table S4. The Pfam domains found in the head and salivary gland transcriptome.

Additional file 6: Table S5. Proteins with interesting functions annotated to Pfam and Nr database.

Additional file 7: Table S6. List of proteins functionally annotated to Nr database but with no Pfam domain.

Additional file 8: Table S7. Annotation summary of potential effector proteins (based of Arabidopsis fed *S. litura*).

Additional file 9: Table S8. Primers used for RT-PCR of highly expressed genes in head and salivary gland.

Additional file 10: Figure R1-R17. Raw and unprocessed gel pictures corresponding to Fig 9

Figure S1

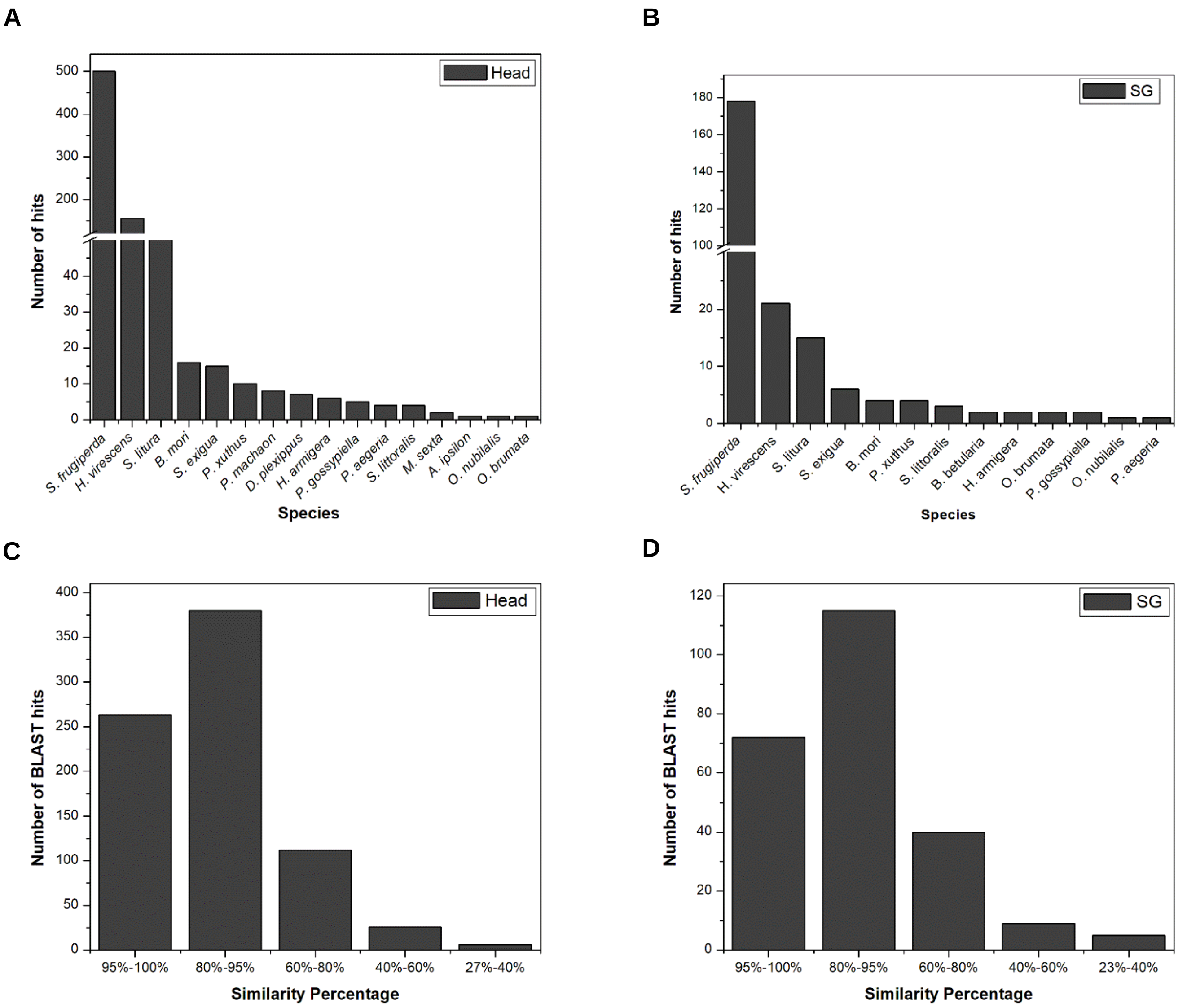
A
Head
Number of hits
500
400
300
200
40
30
20
10
0
S. frugiperda
H. virescens
S. litura
B. mori
S. exigua
P. xuthus
P. machaon
D. plexippus
H. armigera
P. gossypiella
P. aegeria
S. littoralis
M. sexta
A. ipsilon
O. nubilalis
O. brumata
Species
B
SG
Number of hits
180
160
140
120
100
20
10
0
S. frugiperda
H. virescens
S. litura
S. exigua
B. mori
P. xuthus
S. littoralis
B. betularia
H. armigera
O. brumata
P. gossypiella
O. nubilalis
P. aegeria
Species
C
Head
Number of BLAST hits
400
350
300
250
200
150
100
50
0
95%-100%
80%-95%
60%-80%
40%-60%
27%-40%
Similarity Percentage
D
SG
Number of BLAST hits
120
100
80
60
40
20
0
95%-100%
80%-95%
60%-80%
40%-60%
23%-40%
Similarity Percentage

Figure S2

A

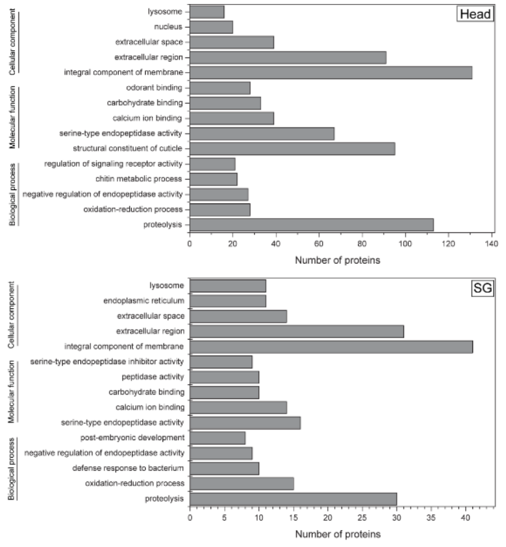
Head
Cellular component
lysosome
nucleus
extracellular space
extracellular region
integral component of membrane
Molecular function
odorant binding
carbohydrate binding
calcium ion binding
serine-type endopeptidase activity
structural constituent of cuticle
Biological process
regulation of signaling receptor activity
chitin metabolic process
negative regulation of endopeptidase activity
oxidation-reduction process
proteolysis
0 20 40 60 80 100 120 140
Number of proteins
SG
Cellular component
lysosome
endoplasmic reticulum
extracellular space
extracellular region
integral component of membrane
Molecular function
serine-type endopeptidase inhibitor activity
peptidase activity
carbohydrate binding
calcium ion binding
serine-type endopeptidase activity
Biological process
post-embryonic development
negative regulation of endopeptidase activity
defense response to bacterium
oxidation-reduction process
proteolysis
0 5 10 15 20 25 30 35 40
Number of proteins

B

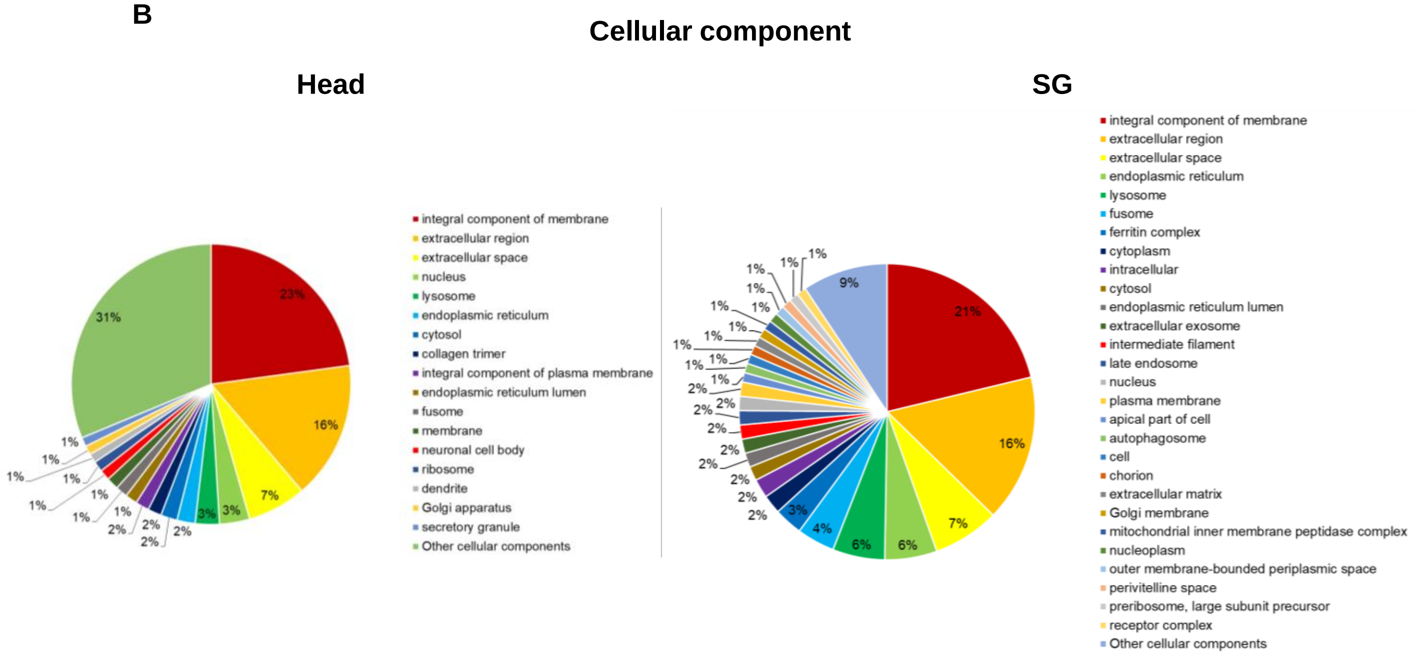
Cellular component
Head
23%
16%
7%
3%
3%
31%
integral component of membrane
extracellular region
extracellular space
nucleus
lysosome
endoplasmic reticulum
cytosol
collagen trimer
integral component of plasma membrane
endoplasmic reticulum lumen
fusome
membrane
neuronal cell body
ribosome
dendrite
Golgi apparatus
secretory granule
Other cellular components
SG
21%
16%
7%
6%
6%
4%
3%
9%
integral component of membrane
extracellular region
extracellular space
endoplasmic reticulum
lysosome
fusome
ferritin complex
cytoplasm
intracellular
cytosol
endoplasmic reticulum lumen
extracellular exosome
intermediate filament
late endosome
nucleus
plasma membrane
apical part of cell
autophagosome
cell
chorion
extracellular matrix
Golgi membrane
mitochondrial inner membrane peptidase complex
nucleoplasm
outer membrane-bounded periplasmic space
perivitelline space
preribosome, large subunit precursor
receptor complex
Other cellular components

Figure S3

A

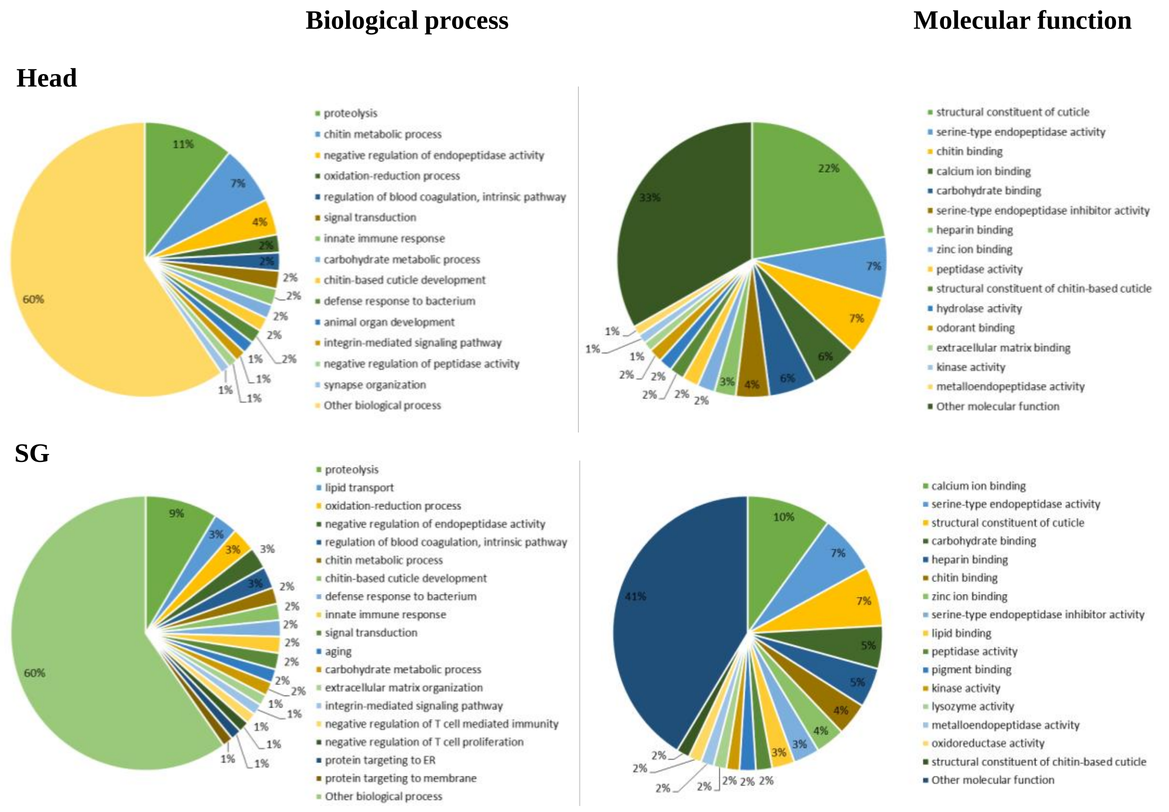

Biological process
Molecular function
Head
proteolysis
chitin metabolic process
negative regulation of endopeptidase activity
oxidation-reduction process
regulation of blood coagulation, intrinsic pathway
signal transduction
innate immune response
carbohydrate metabolic process
chitin-based cuticle development
defense response to bacterium
animal organ development
integrin-mediated signaling pathway
negative regulation of peptidase activity
synapse organization
Other biological process
structural constituent of cuticle
serine-type endopeptidase activity
chitin binding
calcium ion binding
carbohydrate binding
serine-type endopeptidase inhibitor activity
heparin binding
zinc ion binding
peptidase activity
structural constituent of chitin-based cuticle
hydrolase activity
odorant binding
extracellular matrix binding
kinase activity
metalloendopeptidase activity
Other molecular function
SG
proteolysis
lipid transport
oxidation-reduction process
negative regulation of endopeptidase activity
regulation of blood coagulation, intrinsic pathway
chitin metabolic process
chitin-based cuticle development
defense response to bacterium
innate immune response
signal transduction
aging
carbohydrate metabolic process
extracellular matrix organization
integrin-mediated signaling pathway
negative regulation of T cell mediated immunity
negative regulation of T cell proliferation
protein targeting to ER
protein targeting to membrane
Other biological process
calcium ion binding
serine-type endopeptidase activity
structural constituent of cuticle
carbohydrate binding
heparin binding
chitin binding
zinc ion binding
serine-type endopeptidase inhibitor activity
lipid binding
peptidase activity
pigment binding
kinase activity
lysozyme activity
metalloendopeptidase activity
oxidoreductase activity
structural constituent of chitin-based cuticle
Other molecular function

B

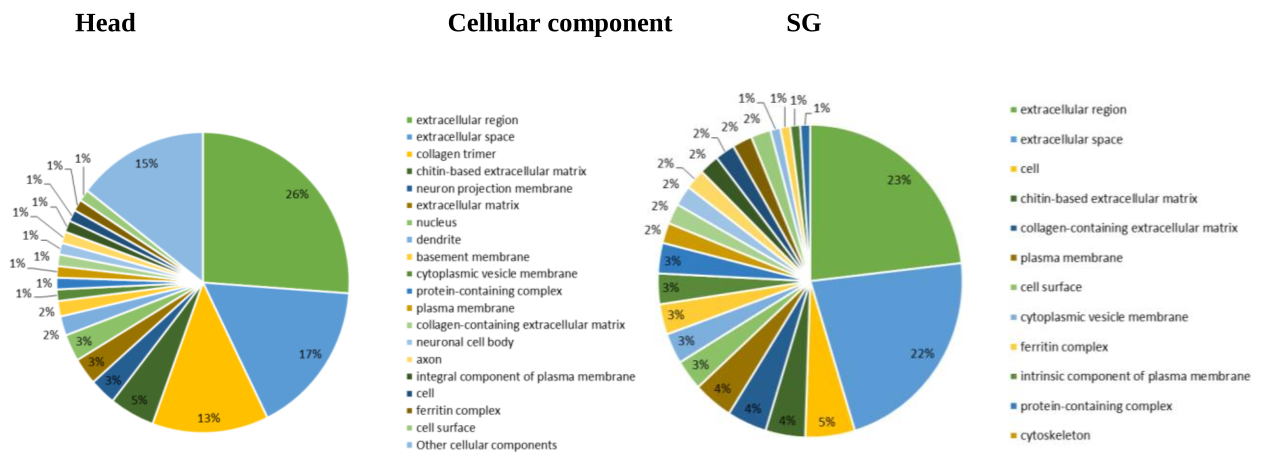

Head
Cellular component
SG
extracellular region
extracellular space
collagen trimer
chitin-based extracellular matrix
neuron projection membrane
extracellular matrix
nucleus
dendrite
basement membrane
cytoplasmic vesicle membrane
protein-containing complex
plasma membrane
collagen-containing extracellular matrix
neuronal cell body
axon
integral component of plasma membrane
cell
ferritin complex
cell surface
Other cellular components
extracellular region
extracellular space
cell
chitin-based extracellular matrix
collagen-containing extracellular matrix
plasma membrane
cell surface
cytoplasmic vesicle membrane
ferritin complex
intrinsic component of plasma membrane
protein-containing complex
cytoskeleton

Figure S4

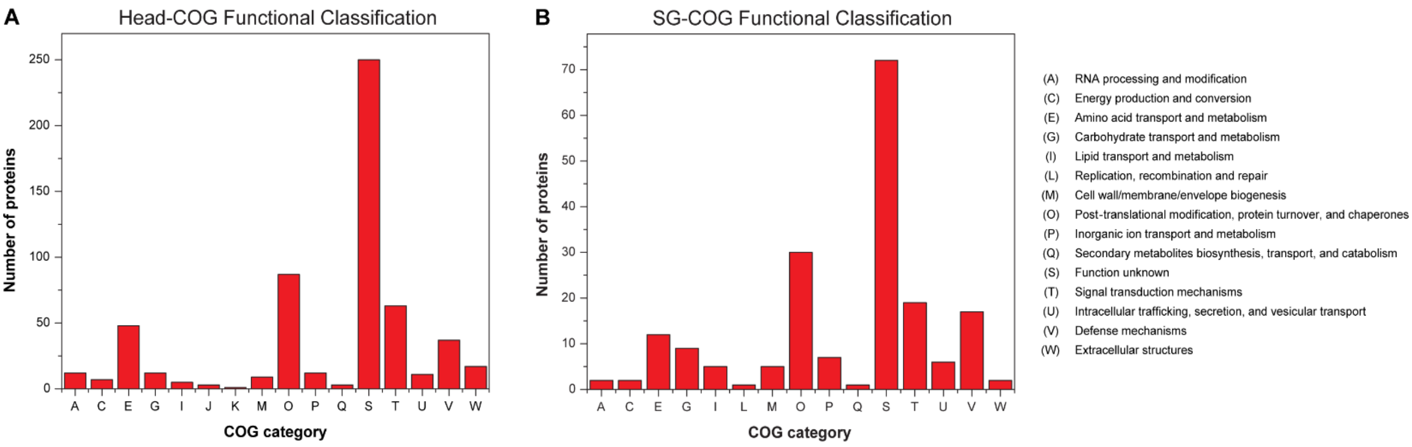
A
Head-COG Functional Classification
Number of proteins
COG category
A C E G I J K M O P Q S T U V W
B
SG-COG Functional Classification
Number of proteins
COG category
A C E G I L M O P Q S T U V W
(A) RNA processing and modification
(C) Energy production and conversion
(E) Amino acid transport and metabolism
(G) Carbohydrate transport and metabolism
(I) Lipid transport and metabolism
(L) Replication, recombination and repair
(M) Cell wall/membrane/envelope biogenesis
(O) Post-translational modification, protein turnover, and chaperones
(P) Inorganic ion transport and metabolism
(Q) Secondary metabolites biosynthesis, transport, and catabolism
(S) Function unknown
(T) Signal transduction mechanisms
(U) Intracellular trafficking, secretion, and vesicular transport
(V) Defense mechanisms
(W) Extracellular structures

Figure S5

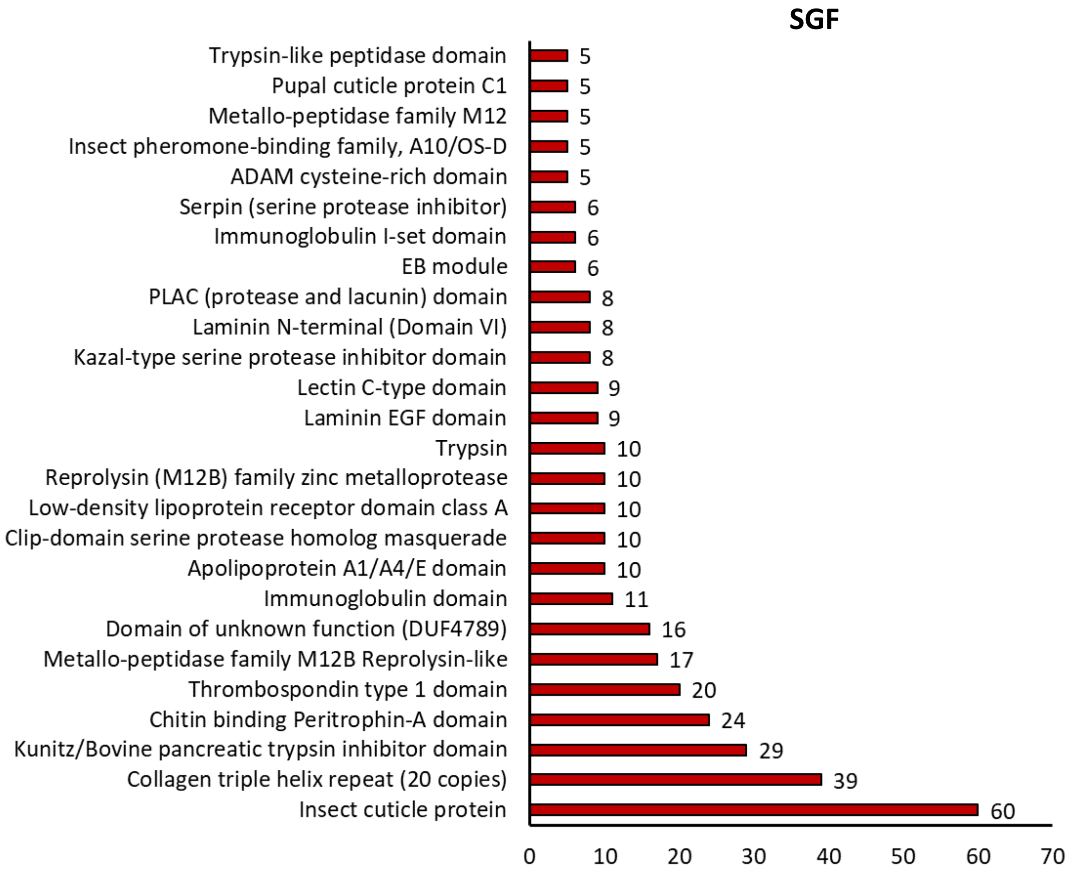
SGF
Trypsin-like peptidase domain
Pupal cuticle protein C1
Metallo-peptidase family M12
Insect pheromone-binding family, A10/OS-D
ADAM cysteine-rich domain
Serpin (serine protease inhibitor)
Immunoglobulin I-set domain
EB module
PLAC (protease and lacunin) domain
Laminin N-terminal (Domain VI)
Kazal-type serine protease inhibitor domain
Lectin C-type domain
Laminin EGF domain
Trypsin
Reprolysin (M12B) family zinc metalloprotease
Low-density lipoprotein receptor domain class A
Clip-domain serine protease homolog masquerade
Apolipoprotein A1/A4/E domain
Immunoglobulin domain
Domain of unknown function (DUF4789)
Metallo-peptidase family M12B Reprolysin-like
Thrombospondin type 1 domain
Chitin binding Peritrophin-A domain
Kunitz/Bovine pancreatic trypsin inhibitor domain
Collagen triple helix repeat (20 copies)
Insect cuticle protein
5
5
5
5
5
6
6
6
8
8
8
9
9
10
10
10
10
10
11
16
17
20
24
29
39
60
0
10
20
30
40
50
60
70
