# Supplementary material for: In silico identification of effector proteins from generalist herbivore Spodoptera litura
Source: BMC Genomics. 2020 Nov 23;21:819. doi: 10.1186/s12864-020-07196-4 (PMC7681983; doi:10.1186/s12864-020-07196-4)
Supplement: Supplementary file 10 — Additional file 10: Figure R1-R17. Raw and unprocessed gel pictures correspondingto Fig. 9. [file 12864_2020_7196_MOESM10_ESM.pdf]

Figure R1

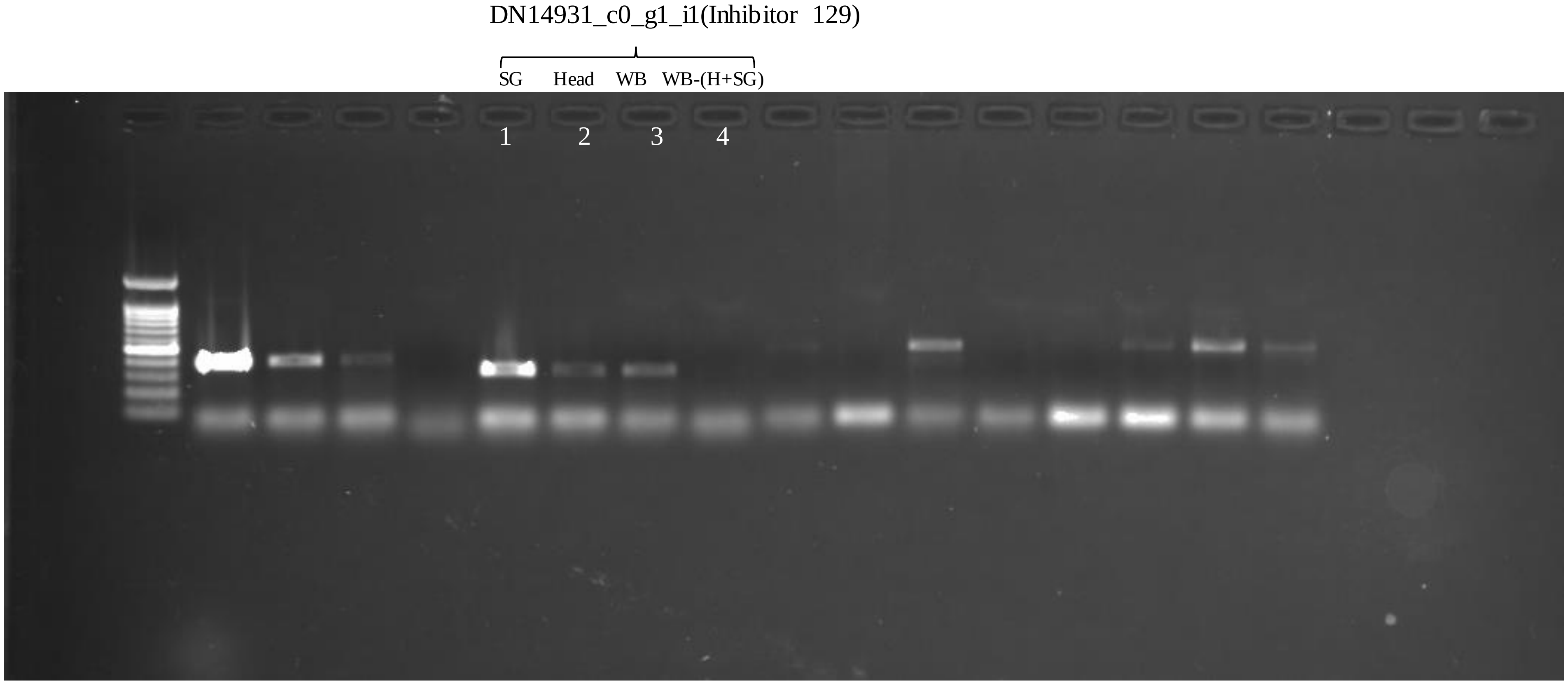

Figure R1: RT-PCR amplification of potential effector encoding gene DN14931_c0_g1_i1(Inhibitor). The expression is checked in different tissues, SG (salivary gland), WB (whole body of insect), WB-(H+SG) (whole body without head and salivary gland). PCR samples were loaded into different wells denoted by numbers 1-4.

Figure R2

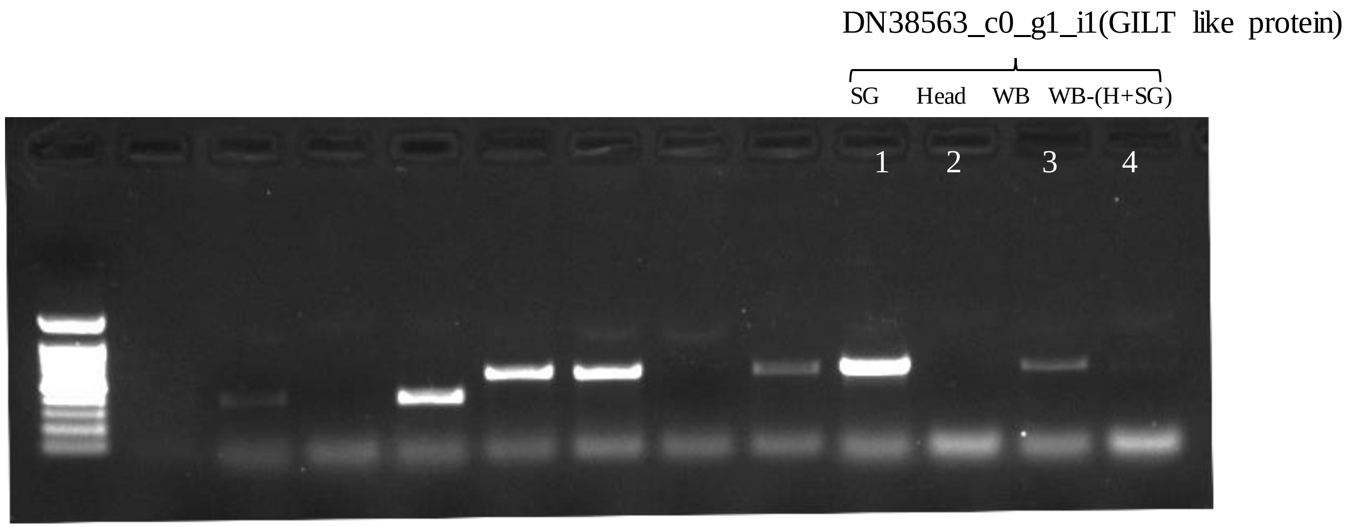

Figure R2: RT-PCR amplification of potential effector encoding gene DN38563_c0_g1_i1(GILT like protein). The expression is checked in different tissues, SG (salivary gland), WB (whole body of insect), WB-(H+SG) (whole body without head and salivary gland). PCR samples were loaded into different wells denoted by numbers 1-4.

Figure R3

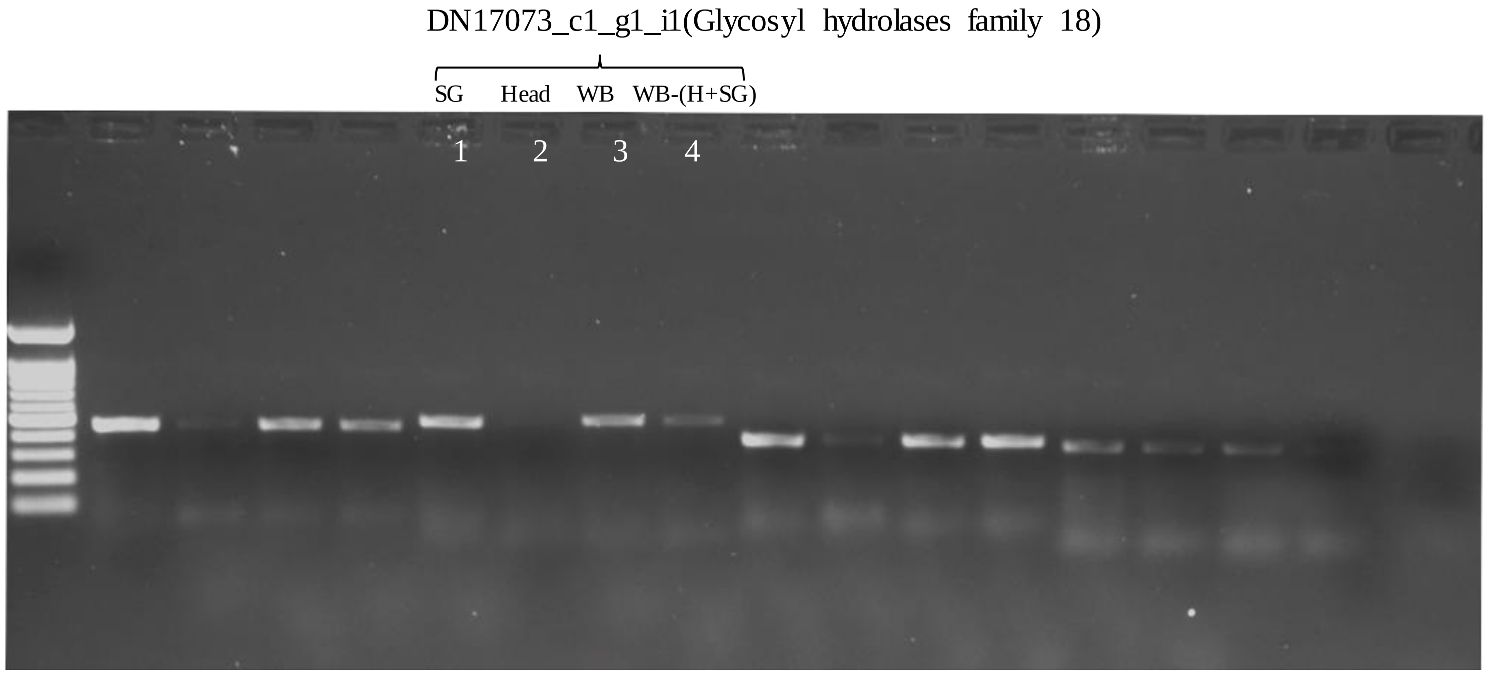

Figure R3: RT-PCR amplification of potential effector encoding gene DN17073_c1_g1_i1(Glycosyl hydrolases family 18). The expression is checked in different tissues, SG (salivary gland), WB (whole body of insect), WB-(H+SG) (whole body without head and salivary gland). PCR samples were loaded into different wells denoted by numbers 1-4.

Figure R4

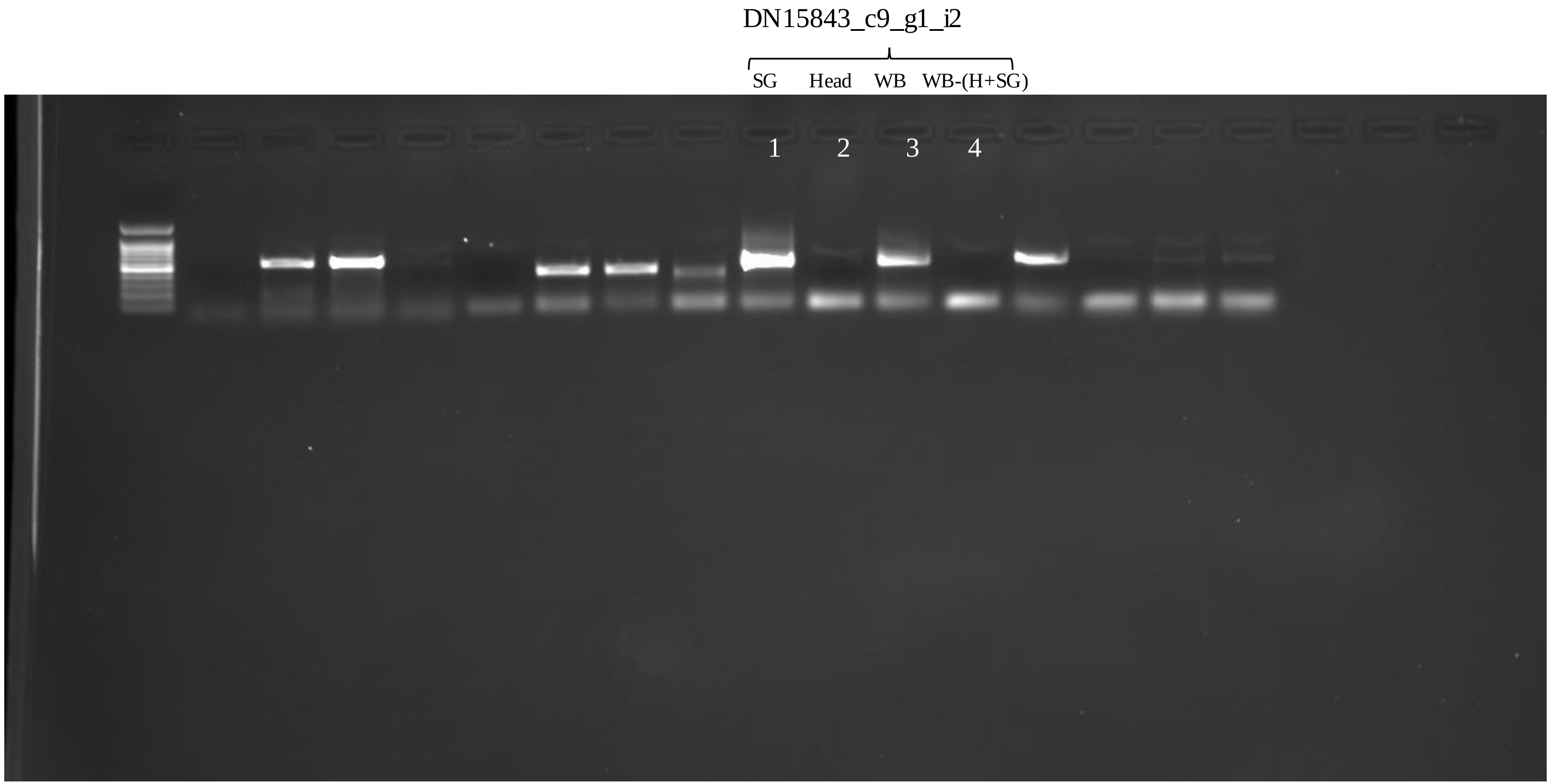

Figure R4: RT-PCR amplification of potential effector encoding gene DN15843_c9_g1_i2. The expression is checked in different tissues, SG (salivary gland), WB (whole body of insect), WB-(H+SG) (whole body without head and salivary gland). PCR samples were loaded into different wells denoted by numbers 1-4.

Figure R5

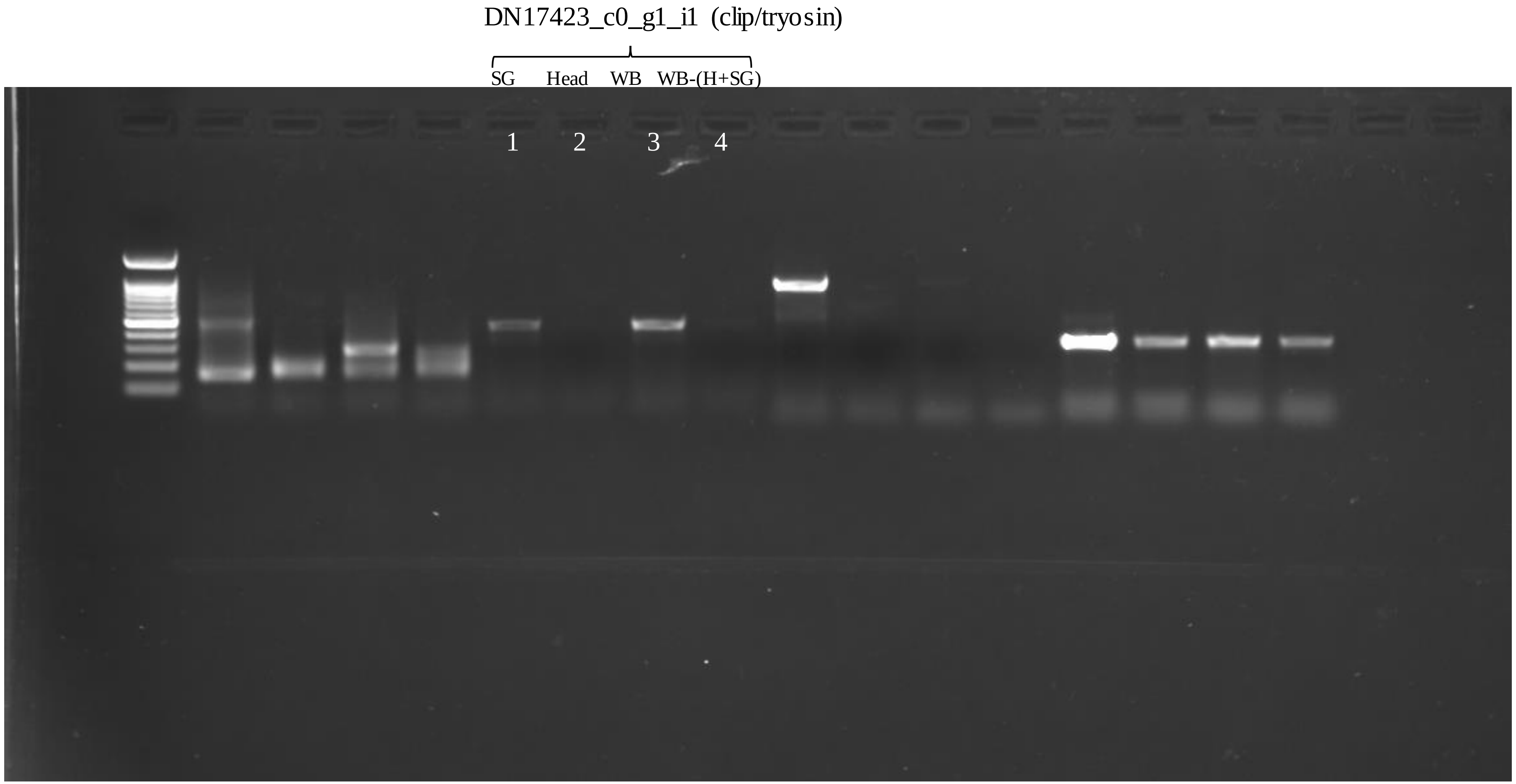

Figure R5: RT-PCR amplification of potential effector encoding gene DN17423_c0_g1_i1 (clip/tryosin). The expression is checked in different tissues, SG (salivary gland), WB (whole body of insect), WB-(H+SG) (whole body without head and salivary gland). PCR samples were loaded into different wells denoted by numbers 1-4.

Figure R6

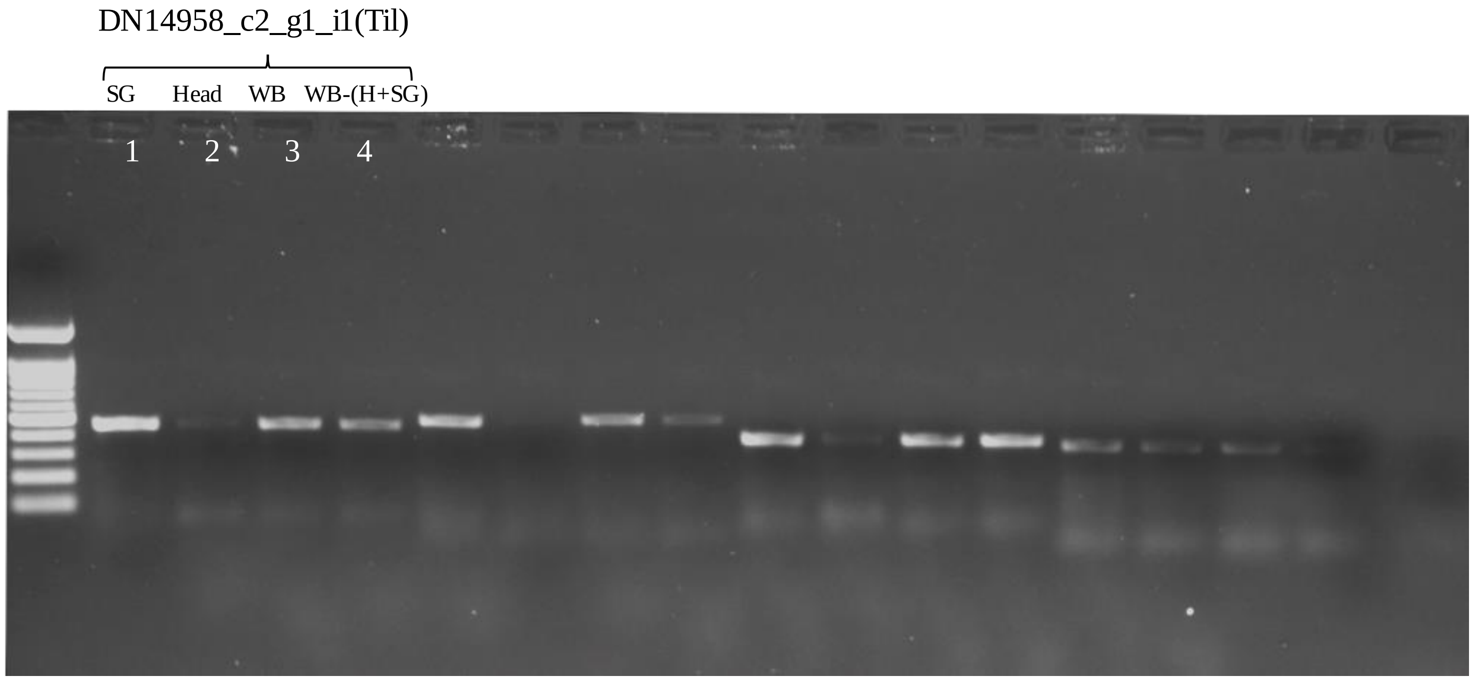

Figure R6: RT-PCR amplification of potential effector encoding gene DN14958_c2_g1_i1(Til). The expression is checked in different tissues, SG (salivary gland), WB (whole body of insect), WB-(H+SG) (whole body without head and salivary gland). PCR samples were loaded into different wells denoted by numbers 1-4.

Figure R7

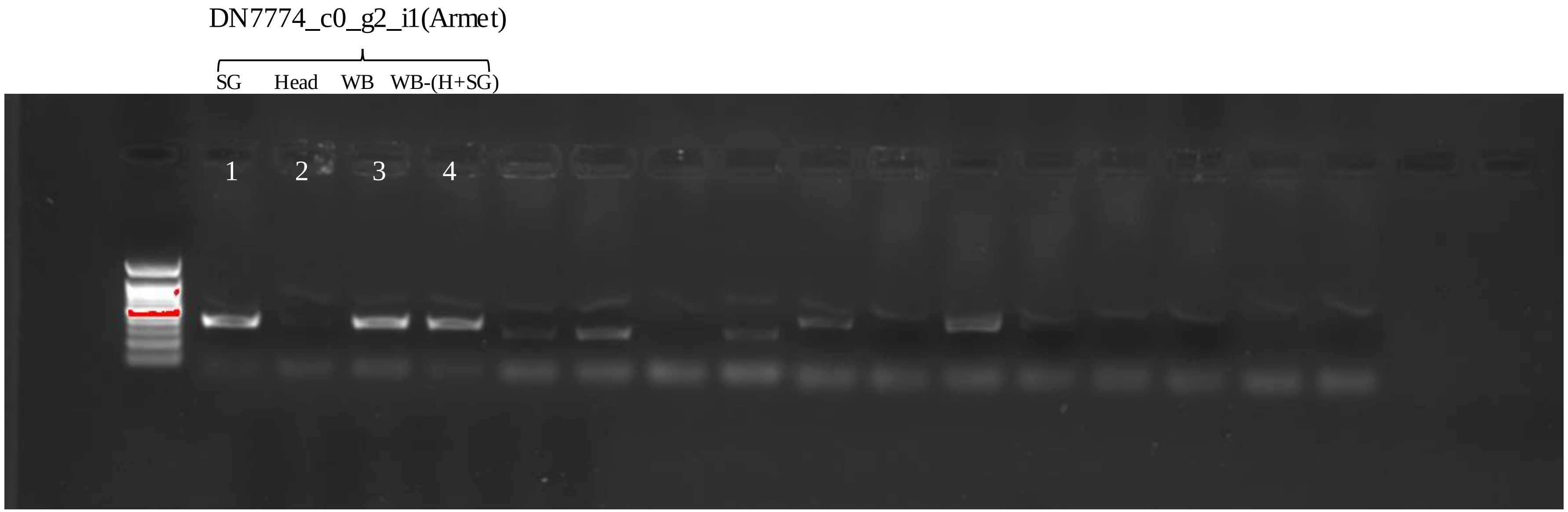

Figure R7: RT-PCR amplification of potential effector encoding gene DN7774_c0_g2_i1(Armet). The expression is checked in different tissues, SG (salivary gland), WB (whole body of insect), WB-(H+SG) (whole body without head and salivary gland). PCR samples were loaded into different wells denoted by numbers 1-4.

Figure R8

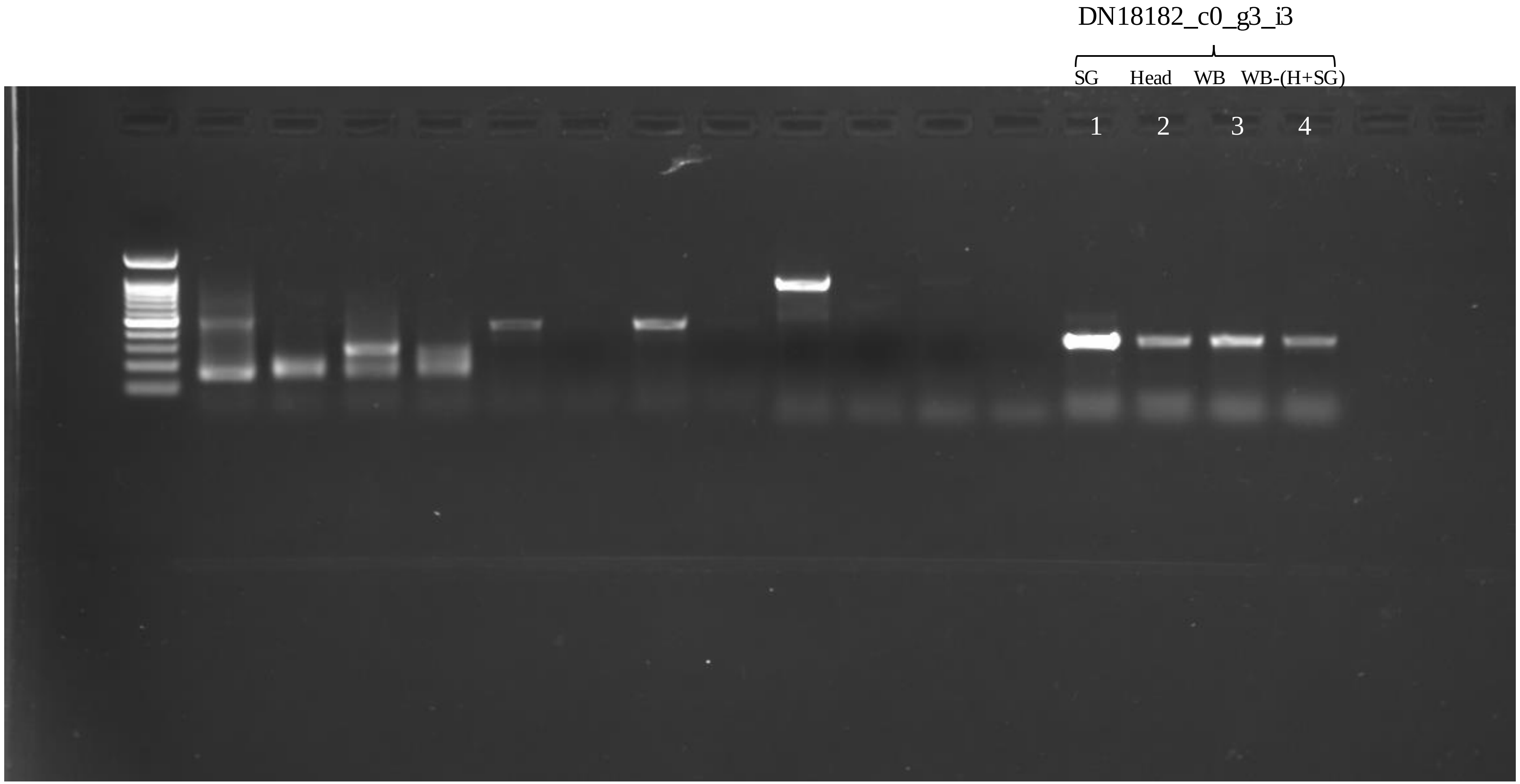

Figure R8: RT-PCR amplification of potential effector encoding gene DN18182_c0_g3_i3. The expression is checked in different tissues, SG (salivary gland), WB (whole body of insect), WB-(H+SG) (whole body without head and salivary gland). PCR samples were loaded into different wells denoted by numbers 1-4.

Figure R9

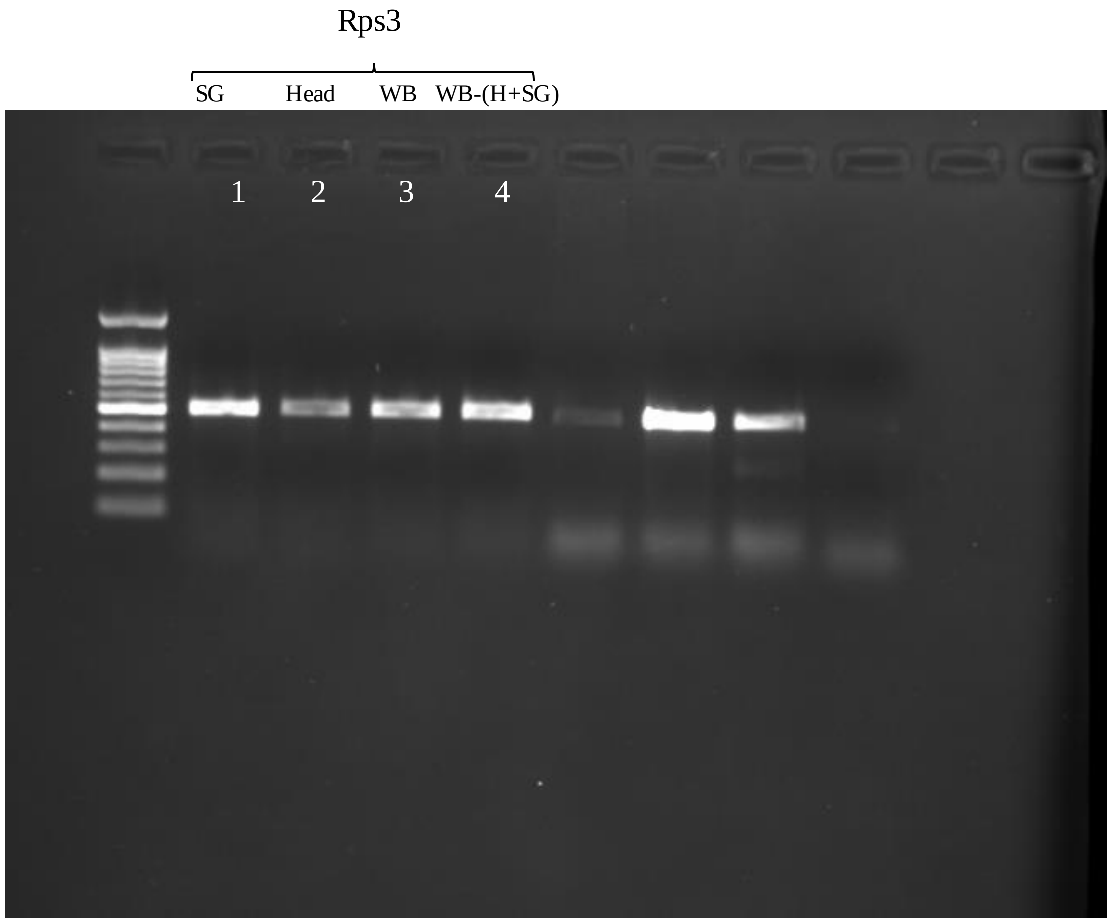

Figure R9: RT-PCR amplification of insect house keeping encoding gene *RPS3*. The expression is checked in different tissues, SG (salivary gland), WB (whole body of insect), WB-(H+SG) (whole body without head and salivary gland). PCR samples were loaded into different wells denoted by numbers 1-4.

Figure R10

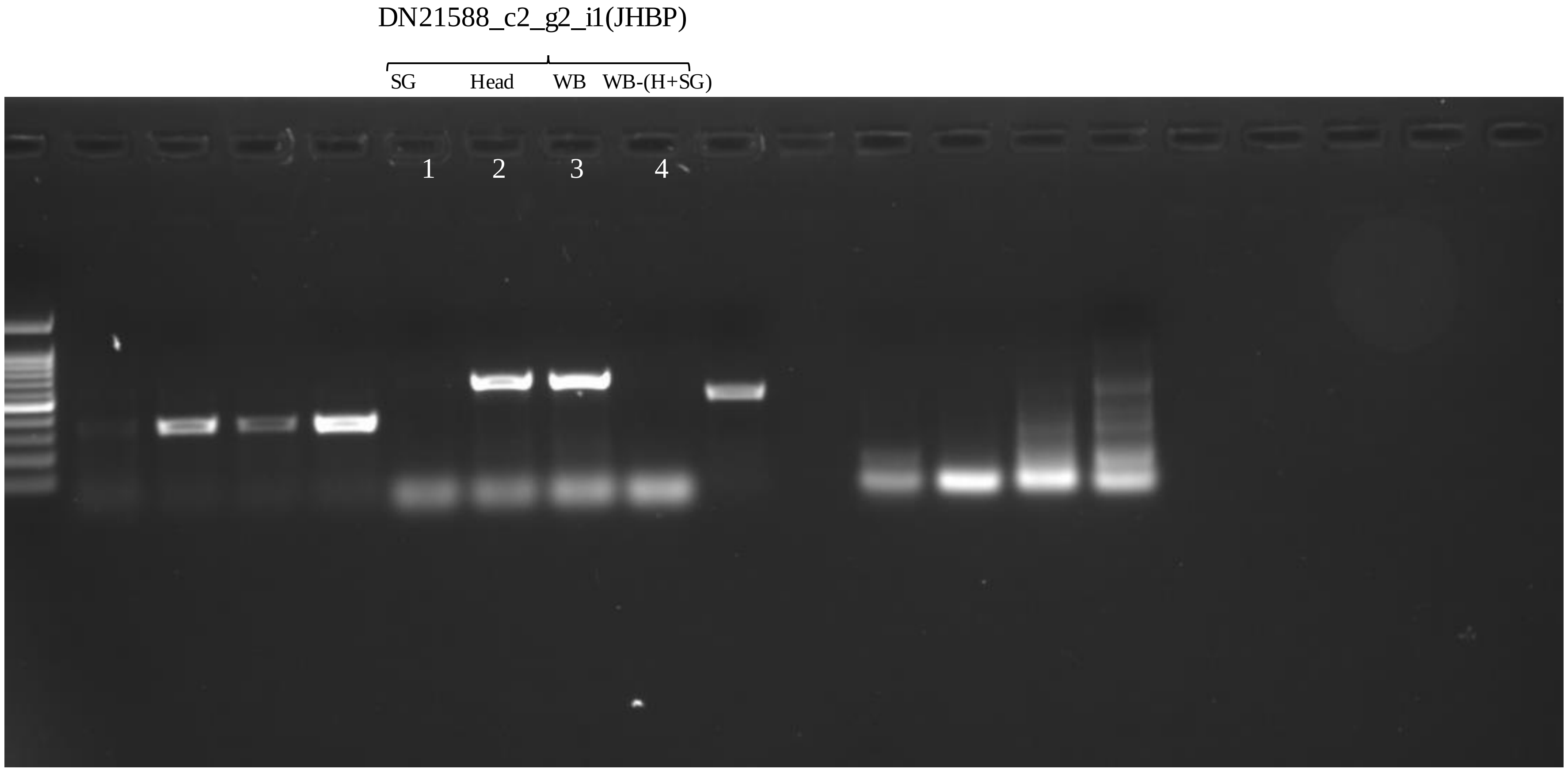

Figure R10: RT-PCR amplification of potential effector encoding gene DN21588_c2_g2_i1(JHBP). The expression is checked in different tissues, SG (salivary gland), WB (whole body of insect), WB-(H+SG) (whole body without head and salivary gland). PCR samples were loaded into different wells denoted by numbers 1-4.

Figure R11

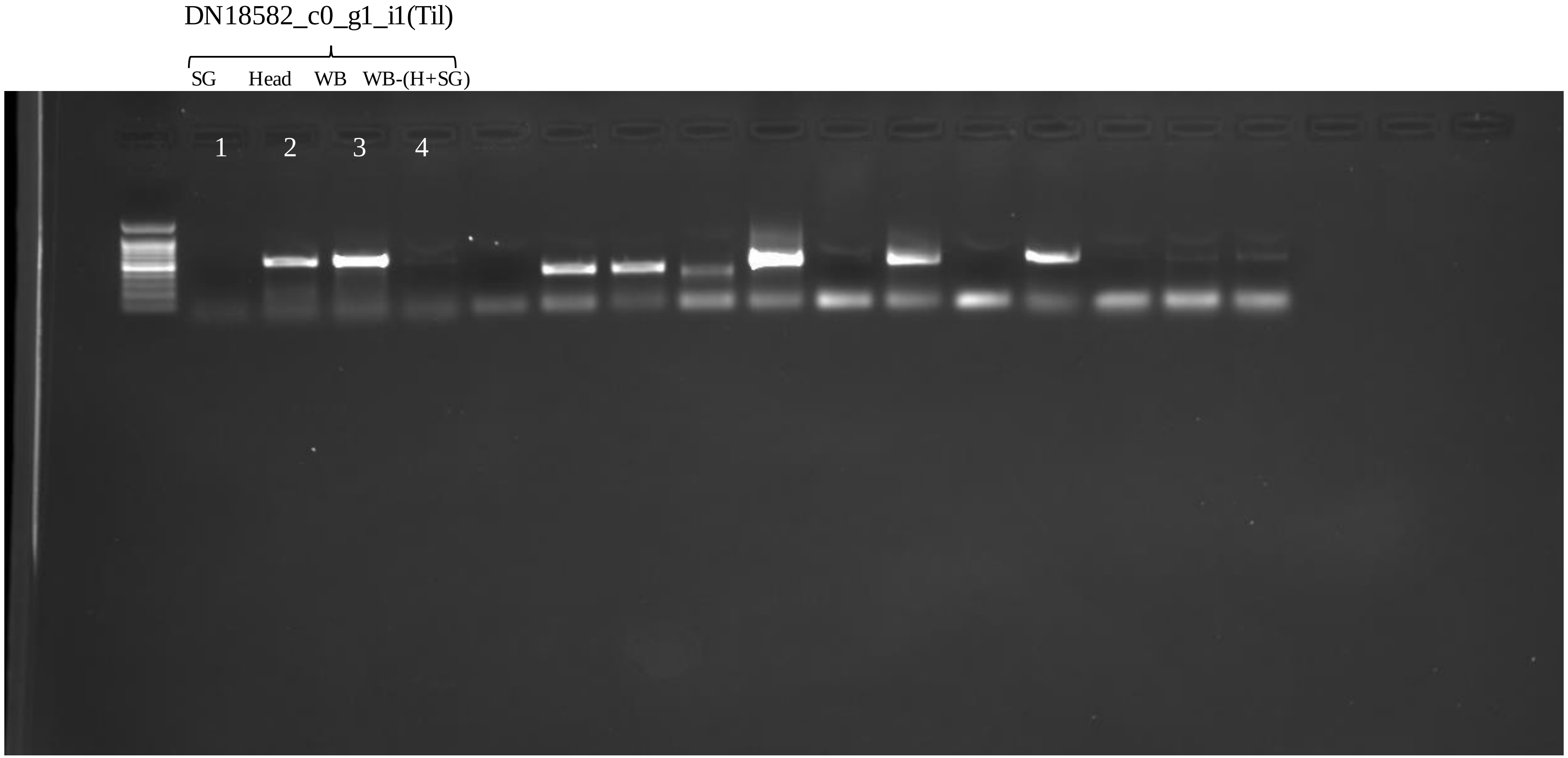

Figure R11: RT-PCR amplification of potential effector encoding gene DN18582_c0_g1_i1(Til)). The expression is checked in different tissues, SG (salivary gland), WB (whole body of insect), WB-(H+SG) (whole body without head and salivary gland). PCR samples were loaded into different wells denoted by numbers 1-4.

Figure R12

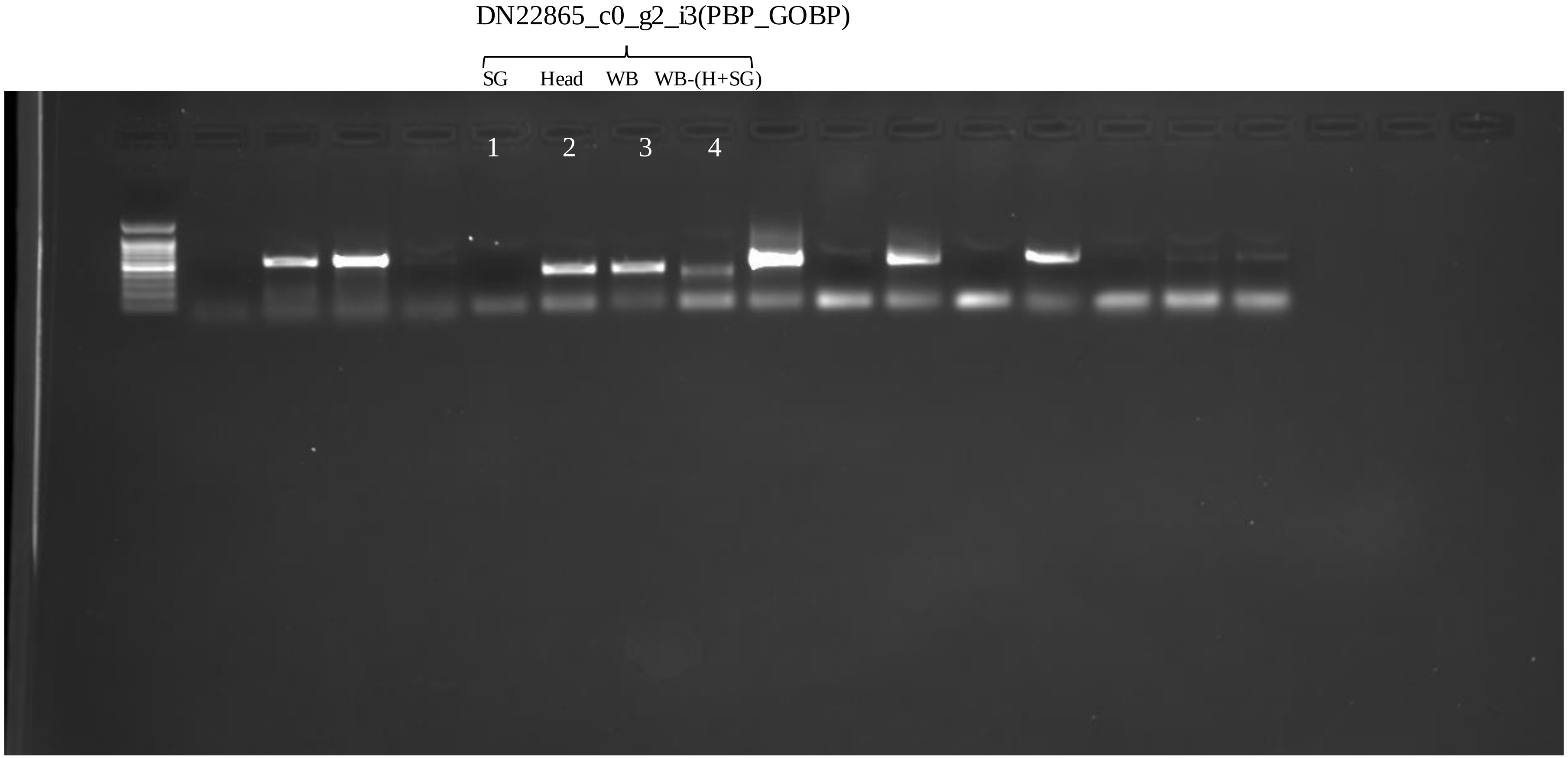

Figure R12: RT-PCR amplification of potential effector encoding gene DN22865_c0_g2_i3(PBP_GOBP). The expression is checked in different tissues, SG (salivary gland), WB (whole body of insect), WB-(H+SG) (whole body without head and salivary gland). PCR samples were loaded into different wells denoted by numbers 1-4.

Figure R13

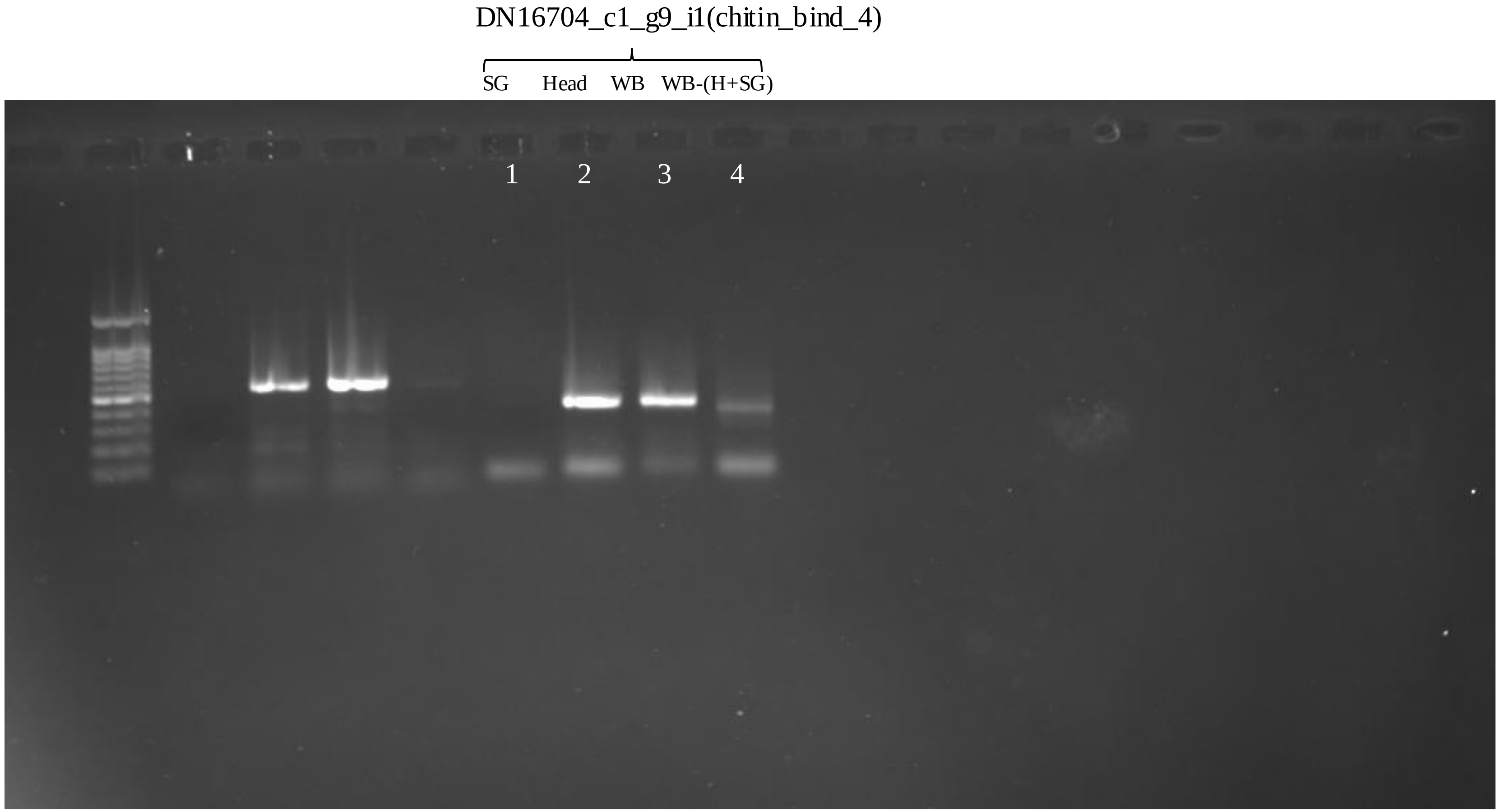

Figure R13: RT-PCR amplification of potential effector encoding gene DN16704_c1_g9_i1(chitin_bind_4). The expression is checked in different tissues, SG (salivary gland), WB (whole body of insect), WB-(H+SG) (whole body without head and salivary gland). PCR samples were loaded into different wells denoted by numbers 1-4.

Figure R14

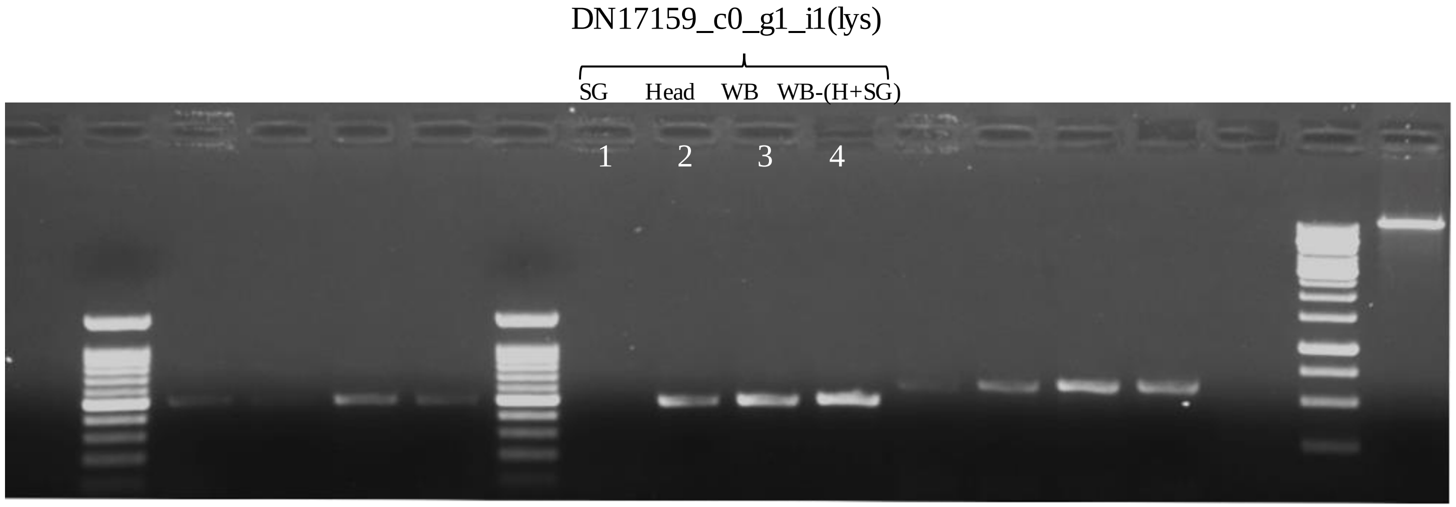

Figure R14: RT-PCR amplification of potential effector encoding gene DN17159_c0_g1_i1(lys). The expression is checked in different tissues, SG (salivary gland), WB (whole body of insect), WB-(H+SG) (whole body without head and salivary gland). PCR samples were loaded into different wells denoted by numbers 1-4.

Figure R15

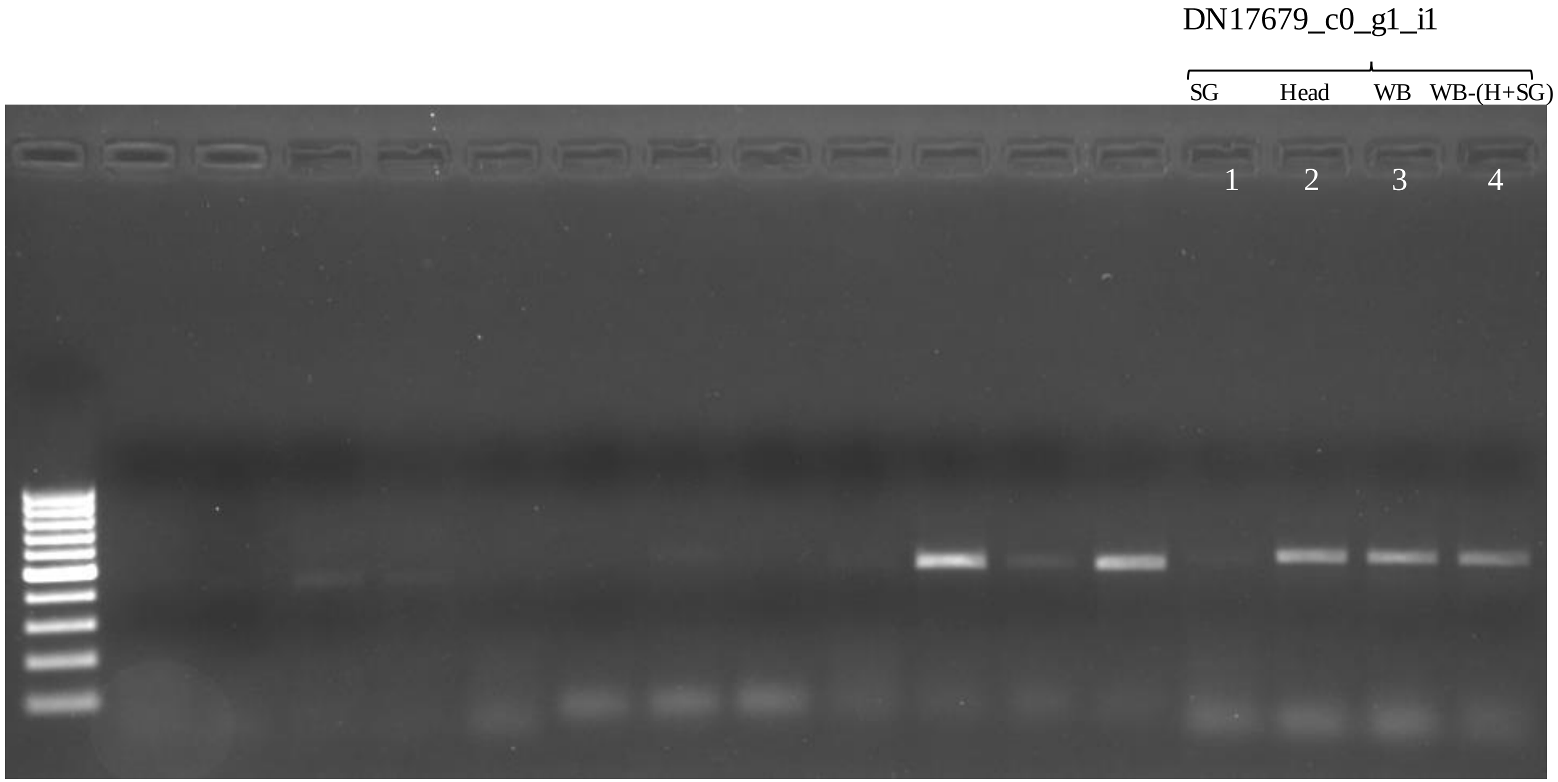

Figure R15: RT-PCR amplification of potential effector encoding gene DN17679_c0_g1_i1). The expression is checked in different tissues, SG (salivary gland), WB (whole body of insect), WB-(H+SG) (whole body without head and salivary gland). PCR samples were loaded into different wells denoted by numbers 1-4.

Figure R16

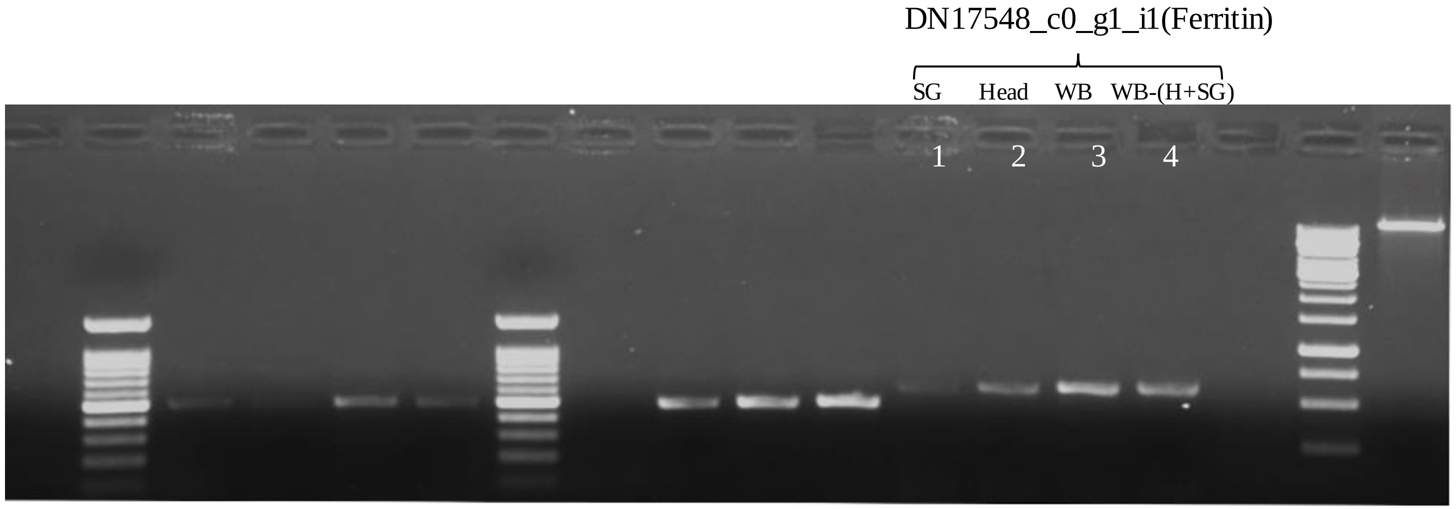

Figure R16: RT-PCR amplification of potential effector encoding gene DN17548_c0_g1_i1(Ferritin). The expression is checked in different tissues, SG (salivary gland), WB (whole body of insect), WB-(H+SG) (whole body without head and salivary gland). PCR samples were loaded into different wells denoted by numbers 1-4.

Figure R17

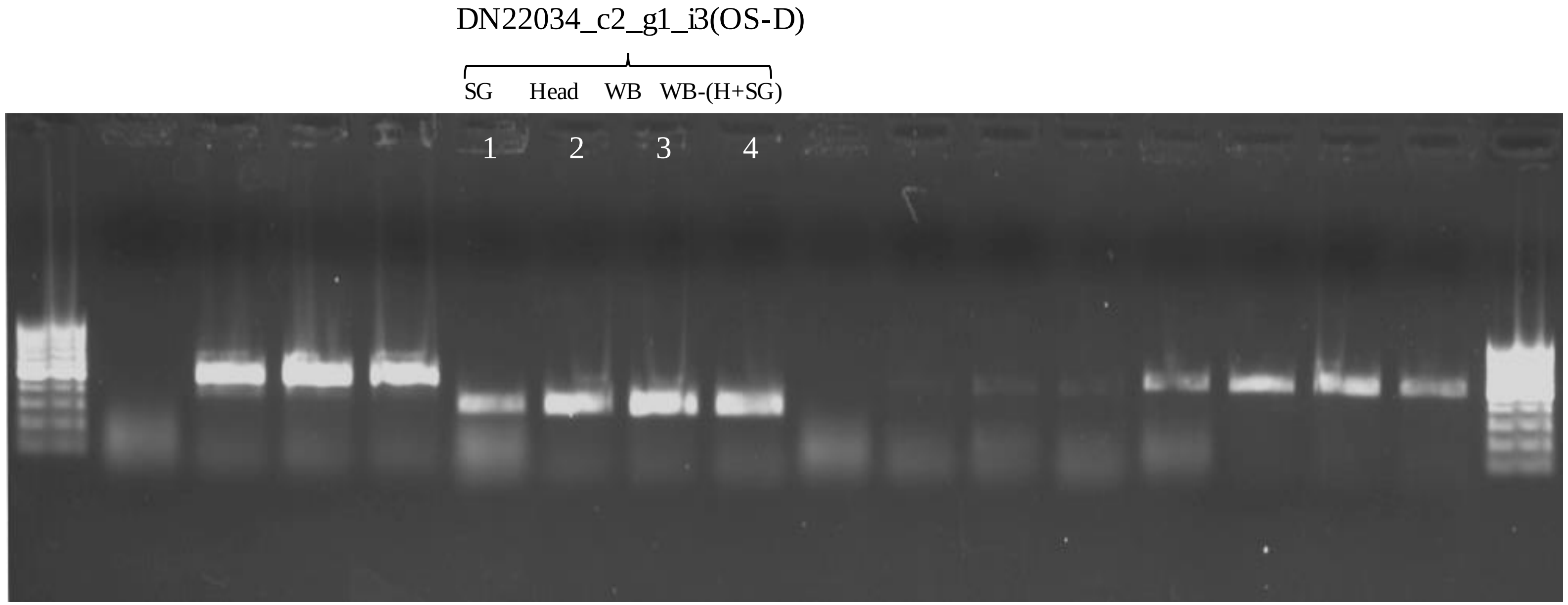

Figure R17: RT-PCR amplification of potential effector encoding gene DN22034_c2_g1_i3(OS-D). The expression is checked in different tissues, SG (salivary gland), WB (whole body of insect), WB-(H+SG) (whole body without head and salivary gland). PCR samples were loaded into different wells denoted by numbers 1-4.
